# Predicting novel mosquito-associated viruses from metatranscriptomic dark matter

**DOI:** 10.1093/nargab/lqae077

**Published:** 2024-07-02

**Authors:** Amanda Araújo Serrão de Andrade, Otávio Brustolini, Marco Grivet, Carlos G Schrago, Ana Tereza Ribeiro Vasconcelos

**Affiliations:** Bioinformatics Laboratory (LABINFO), National Laboratory for Scientific Computing, Petrópolis 25651-076, Brazil; Bioinformatics Laboratory (LABINFO), National Laboratory for Scientific Computing, Petrópolis 25651-076, Brazil; Pontifical Catholic University of Rio de Janeiro, Rio de Janeiro 22453-900, Brazil; Federal University of Rio de Janeiro, Rio de Janeiro 21941-913, Brazil; Bioinformatics Laboratory (LABINFO), National Laboratory for Scientific Computing, Petrópolis 25651-076, Brazil

## Abstract

The exponential growth of metatranscriptomic studies dedicated to arboviral surveillance in mosquitoes has yielded an unprecedented volume of unclassified sequences referred to as the virome dark matter. Mosquito-associated viruses are classified based on their host range into Mosquito-specific viruses (MSV) or Arboviruses. While MSV replication is restricted to mosquito cells, Arboviruses infect both mosquito vectors and vertebrate hosts. We developed the MosViR pipeline designed to identify complex genomic discriminatory patterns for predicting novel MSV or Arboviruses from viral contigs as short as 500 bp. The pipeline combines the predicted probability score from multiple predictive models, ensuring a robust classification with Area Under ROC (AUC) values exceeding 0.99 for test datasets. To assess the practical utility of MosViR in actual cases, we conducted a comprehensive analysis of 24 published mosquito metatranscriptomic datasets. By mining this metatranscriptomic dark matter, we identified 605 novel mosquito-associated viruses, with eight putative novel Arboviruses exhibiting high probability scores. Our findings highlight the limitations of current homology-based identification methods and emphasize the potentially transformative impact of the MosViR pipeline in advancing the classification of mosquito-associated viruses. MosViR offers a powerful and highly accurate tool for arboviral surveillance and for elucidating the complexities of the mosquito RNA virome.

## Graphical Abstract

**Graphical Abstract:**
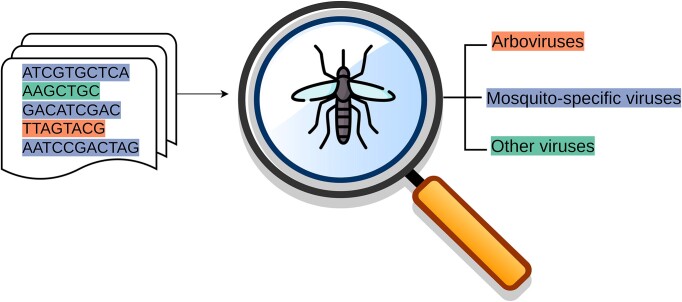

## Introduction

Mosquito-borne pathogens significantly contribute to disease outbreaks and epidemics, leading to high mortality and morbidity rates in both human and animal populations (1). The rising temperatures, urbanization and increased trade have expanded the geographic range of multiple mosquito species, placing 80% of the global population at risk of infection by mosquito-borne pathogens (2). This growing worldwide concern has intensified virome surveillance of mosquitoes for early pathogen identification, enhancing public health preparedness for disease outbreaks (3). These surveillance efforts have revealed an unprecedented viral diversity within mosquitoes, uncovering newly emerged mosquito-associated viruses (4,5).

The expanding landscape of mosquito-associated viruses encompasses Arthropod-borne viruses (Arboviruses) and Mosquito-specific viruses (MSV). Arboviruses are associated with high-burden human diseases such as hemorrhagic fevers and encephalitis and present dual-host tropism as they cycle between vertebrate hosts and mosquito vectors (6). MSVs encompass 98–99% of the mosquito virome (7). These viruses present host restriction to infect mosquito populations, causing a persistent viral infection maintained vertically in nature (7,8). MSV host restriction may occur at different stages of the replication cycle, involving genetic elements (structural and non-structural genes and untranslated terminal regions), vertebrate host antiviral immune responses, and the temperature-dependent microenvironment (9,10). Although these MSVs are non-pathogenic to humans, they can act as biocontrol agents by modulating the replication of arboviruses. An important biocontrol mechanism is superinfection exclusion, where a primary virus infection prevents the replication of a coinfecting phylogenetically closely related virus (11,12). The frequent coinfection and the co-evolutionary relationship between these viruses suggest that MSV may be ancestors of arboviruses with an expanded host range to include vertebrates (9,10).

Mosquito-associated viral species present a vast genetic diversity, predominantly featuring RNA as their genetic material and exhibiting either linear or segmented genomes. These viruses span various families, such as Flaviviridae, Togaviridae, Peribunyaviridae, Phenuiviridae, Rhabdoviridae, Tymoviridae, Reoviridae and Orthomyxoviridae. Newly identified mosquito-associated viruses belong to diverse RNA virus groups, encompassing viral families previously associated with mosquitoes and those that are not. These include Partitiviridae, Iflaviridae, Dicistroviridae, Totiviridae, Chrysoviridae, and Birnaviridae (13,14). Recently discovered mosquito-associated viruses led to identification of novel viral families and genera. For instance, the Mesoniviridae family and the taxons Chuvirus, Negevirus and Qinvirus (13,15). Some MSVs are closely related to medically relevant arboviruses, while other MSVs are closer to plant or fungal viruses (16).

Mosquito virome surveillance through metatranscriptomics (total RNA sequencing) has revolutionized the discovery of RNA viruses and arboviral surveillance (4,15). This technique enables non-targeted, high-throughput detection of known and novel mosquito-associated viruses from environmental samples, mosquito traps, and animal tissues (4,17). Bioinformatics tools play a crucial role in for assembling and identifying viral sequences, impacting the sensitivity and specificity of metatranscriptomic as a surveillance tool (4). Viral identification typically relies on homology searches against pre-existing sequence databases. However, even when using large public databases, the identification is limited by the diversity of previously annotated viral genomes (4,14,17). For mosquito-associated viral identification, the fundamental disadvantage of the homology-based approach is the higher number of genomes representing arboviruses when compared to MSV. This bias can hinder the discovery of divergent or distantly related mosquito-associated viruses that are often unclassified. These unclassified viruses are known as the virome's dark matter, accounting for 40–90% of all metatranscriptomic sequences. Moreover, most newly discovered mosquito-associated viruses present low-quality and partially sequenced genomes, leaving their taxonomy, host range, and potential pathogenicity undetermined (18,19).

Classifying mosquito-associated viruses as arboviruses or MSVs relies on viral isolation and subsequent inoculation experiments in vertebrate and mosquito cell lines (20). *In silico* classification depends on taxonomy analysis and phylogenetic inference to assess the evolutionary distance between a novel virus and a well-described antigenic complex (21). Since exogenous viruses should carry a protein to replicate their genome, most phylogenetic analyses of newly discovered mosquito-associated viruses focus on the RNA-dependent RNA polymerase (RdRp) protein domain (17,22). The RdRp is highly conserved compared with other RNA virus proteins and is considered the unique hallmark gene present in all RNA viruses (23,24). However, phylogenetic inference poses limitations, particularly with highly divergent novel viruses that may cluster randomly with unrelated lineages. Consequently, this approach has drawbacks to identifying these novel viruses (13,14).

Alignment-free genomic signatures are an alternative to the traditional homology-based method for determining the host range of mosquito-associated viruses (25). These signatures may uncover the genomic compositional signals shaped by natural selection, resulting in adaptation of mosquito-associated viruses with diverse genomic and taxonomic structures to a common host range (26). Previous studies have described genome-level mutations between dual-host and single-host viruses, especially variations in the relative frequencies of dinucleotide composition and codon adaptation index, which is a measure associated with codon usage bias (21,26,27). However, employing these features for classifying mosquito-associated viruses faces substantial challenges, as they are primarily documented for Flaviviridae and might not apply universally across novel viruses from distant families. These features often provide more insights into viral taxonomy rather than host range (28). Consequently, identifying an alignment-free genome trait capable of effectively distinguishing between arboviruses and MSV sequences would significantly enhance viral identification in mosquito surveillance data.

Integrating alignment-free genomic signatures with cutting-edge artificial intelligence (AI) techniques marks a paradigm shift in deciphering intricate discriminatory patterns among viral lineages solely based on host range. Recent advances in machine learning have showcased the potential of using viral and host traits for predictive classifications of novel vectors and hosts for well-known Flaviviruses (29), and the potential transmission of a virus via an arthropod vector, including inference of the specific vector (30). Existing approaches face critical challenges, including a lack of empirical support for novel virus-host associations, potential biases from unbalanced data, increased errors due to the curse of dimensionality, the shorter fragment length obtained for novel viruses identified through metatranscriptomics, as well as phylogenetically structured noise and taxonomic reflection (31). These challenges highlight the difficulty of accurately classifying and characterizing novel viruses, thus hindering advancements in understanding viral diversity and host interactions (28,31).

Here, we introduce the MosViR computational pipeline, designed to process metatranscriptomic mosquito data as input and provide accurate host-based classifications for mosquito-associated viruses from contigs as short as 500 bp. The multiple predictive models used by MosViR accurately classify mosquito-associated viruses into host-based classes: Mosquito-associated viruses, Other viruses, MSVs, and Arboviruses, demonstrating high statistical performance. By applying the MosViR pipeline to publicly available mosquito metatranscriptomic data, our aim is to uncover novel mosquito-associated viruses within the metatranscriptomic dark matter, highlighting the MosViR classification of putative novel viral pathogens overlooked by current viral discovery methods. Our primary goal is to provide researchers an innovative tool that facilitates the classification of unknown contigs by introducing host-based classification to previously unexplored sequences. This holds practical implications, including identifying novel pathogens, detecting potential host shifts, and guidance for the necessity of wet lab experiments. The MosViR pipeline is made available as an R package at https://github.com/labinfo-lncc-br/MosViR.

## Materials and methods

### Data collection

Data collection comprised three stages: (i) selection of viral species, (ii) genome retrieval and fragmentation and (iii) class assignment. Initially, we identified viral species with well-defined taxonomy and host range based on information obtained from Virus-Host (https://www.genome.jp/virushostdb/), International Committee on Taxonomy of Viruses (http://ictv.global), Arboviral Catalog (https://wwwn.cdc.gov/arbocat/), ViralZone (http://viralzone.expasy.org) and from the National Center for Biotechnology Information's (NCBI) Taxonomy (https://ftp.ncbi.nih.gov/pub/taxonomy/). We only selected viral species with RNA genomes (Baltimore classes III, IX and X) and consistent host information across all databases, excluding any species with conflicting or missing data. Supplementary Table S1 provides the compiled information on the selected species.

We retrieved nucleotide sequences from the NCBI RefSeq database for the second data collection stage using esearch and efetch tools (http://eutils.ncbi.nlm.nih.gov). We retained only high-quality complete sequences, excluding those labeled as partial, incomplete, near-complete, or unverified. Removing sequences below 500 bp or exceeding 100 000 bp aimed to improve informational content and computational efficiency. Taxonomy assessment using the taxize R package (https://docs.ropensci.org/taxize/) and the NCBI taxonomy database led to discarding sequences from the unselected viral species. We excluded sequences with >2% non-ACTG bases and curated the remaining dataset at 90% identity using CD-HIT-est (http://weizhong-cluster.ucsd.edu/cd-hit/) to eliminate redundancies. To evaluate the classification performance across different sequence lengths, we fragmented the complete sequences into equal-size fragments of 10 000, 5000, 3000, 1000 and 500 bp using the genocut R package (https://github.com/labinfo-lncc-br/genocut). Each sequence was represented up to three times per fragment length, with no overlapping fragments.

In the concluding stage of data collection, we established our polyphyletic classes based on host range: (i) Mosquito-associated viruses, (ii) Other viruses, being the first splitted into (a) Arboviruses and (b) MSV. Mosquito-associated viruses encompass two sub-categorisation: Arboviruses and MSV. Species labeled as Arboviruses had documented positive cultures in vertebrate cell lines and evidence of an invertebrate vector from the order Diptera (NCBI taxonomy id: 7147). MSV encompasses species that infect only Diptera insects, including vector families such as Culicidae (NCBI taxonomy id: 7157), Ceratopogonidae (NCBI taxonomy id: 41 819), and Psychodidae (NCBI taxonomy id: 7197). Sequences from species without documented relationships with Diptera were labeled as other viruses.

**Figure 1.**
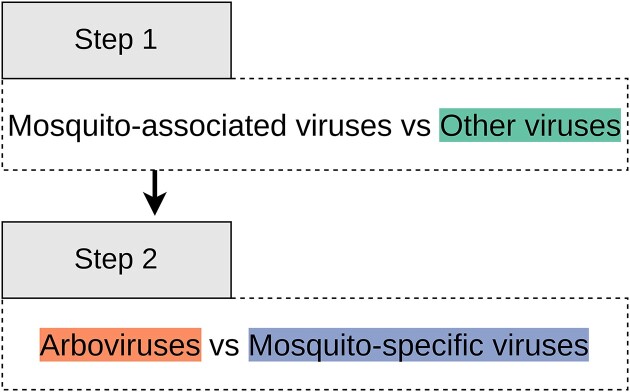
Flowchart illustrating the link between classifiers.

We constructed two sequential binary classifications, the first classification deals with classes: (i) Mosquito-associated viruses and (ii) Other viruses, while the second classification deals with classes: (a) Arboviruses and (b) MSV. A second classifier only considers data classified as Mosquito-associated viruses (Figure 1). The positive classes comprised Mosquito-associated viruses and Arboviruses, while the negative classes had MSVs and Other viruses.

### Feature screening and building single predictive models

To accurately classify the sequences within our polyphyletic classes, we performed a feature-screening analysis considering two alignment-free feature extraction methods: *k*-mer and (*m*,*n*)-mer. These features have demonstrated successful classification of highly divergent viral sequences with short *k*-values (32,33), and were selected based on their classification performance compared to five other alignment-free feature extraction methods, as detailed in Supplementary Material 1. For each fragment length (500, 1000, 3000, 5000 and 10 000 bp), we performed feature extraction with *k* ranging from 2 to 4, to keep the computational cost at a reasonable level. The extraction of *k*-mers and (*m*,*n*)-mers was performed using the mnmer R package with default parameters (33).

Using the createPartition function of the caret package (topepo.github.io/caret/), 70% and 30% of the data were randomly allocated to the training and testing sets, respectively. To address the substantial class imbalance in our datasets, we performed 50 random resamplings of each training set to ensure balanced binary classification with 10 000 instances per class. The selected number of resampled instances was determined based on the number of instances in the smallest dataset. The resampling approach helps mitigate the bias associated with the presence of a majority class during binary classification. In each resampling, we employed a balanced training set to construct a predictive model using the Random Forest (RF) algorithm through the train function of the caret package (topepo.github.io/caret/).

We assess the feature extraction methods based solely on the performance criteria by means of the confusion matrix from each predictive model using the testing sets in the Caret package (topepo.github.io/caret/). We evaluated the metrics F1-Score, Precision, Accuracy, Recall, Sensitivity, Specificity, ROC curve, and Area Under the ROC Curve (AUC) from these matrices. Feature extraction methods were selected based on the highest mean AUC values, with the second choice for those with the highest mean specificity values and smaller values of *k*. High AUC and specificity indicate a lower rate of misclassification for Mosquito-associated viruses and Arboviruses (positive classes), contributing to the overall reliability of the results. By the end of this analysis, we have selected the best-performing feature extraction methods for all fragment lengths considering both classification steps.

### Model selection and soft voting

To ensure optimal pairing of the selected features with the most suitable algorithm, we assessed the performance of six well-established machine-learning algorithms: Support Vector Machine (SVM), K-Nearest Neighbors (KNN), Logistic Regression (LR), RF, Linear Discriminant Analysis (LDA) and Quadratic Discriminant Analysis (QDA). We built 50 predictive models per algorithm for each feature extraction method based on a new random resampling training sets in the caret package (topepo.github.io/caret/). This ensures consistent and fair comparison across different algorithms, enabling a robust evaluation of their performance and meaningful comparison of classification capabilities. We selected the algorithm with the highest AUC as the best classifier for that resampling. Ties are sorted by means of the specificity metric. To identify the best-performing algorithms for each resampling, we repeated this process 50 times per feature matrix (feature set combined with class label assigned to each instance).

To seamlessly transition from individual algorithm evaluations to collective decision-making, we leveraged the best-performing algorithms identified during 50 random resamplings in a soft voting strategy. This mechanism aggregates predictions from diverse machine learning models, improving robustness and accuracy (34,35). This approach enhances the robustness and reliability of the overall classification process by leveraging the strengths of each contributing model to provide comprehensive classification. These carefully curated models will form the core of our MosViR pipeline for classifying mosquito-associated viruses.

### MosViR pipeline

**Figure 2.**
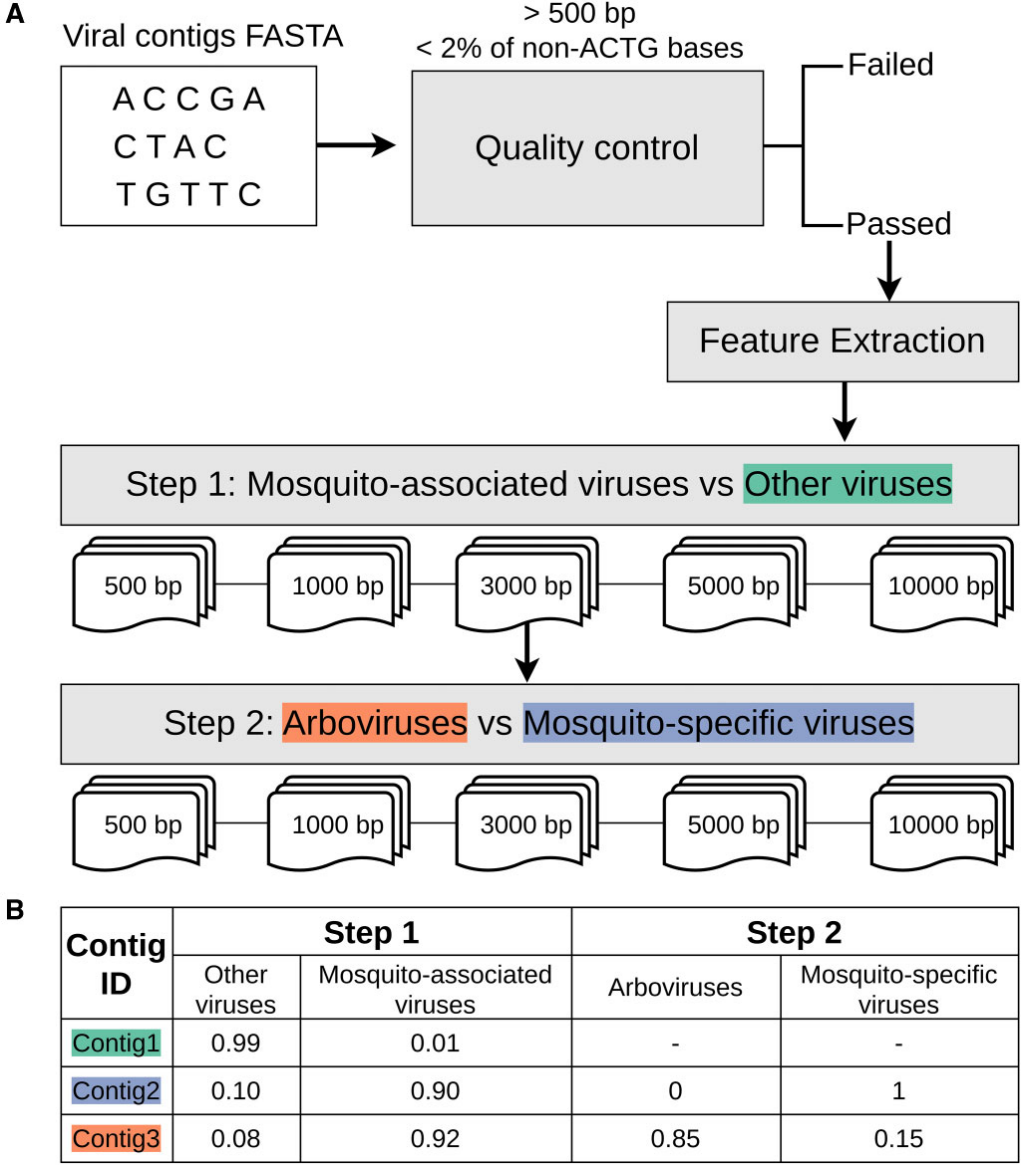
MosViR pipeline. (**A**) Analytical steps included in the pipeline, including the soft voting ensemble built for each fragment length and represented by the paper shades. (**B**) Example of the pipeline output containing the probability scores.

The MosViR pipeline is released as an R package available at GitHub (https://github.com/labinfo-lncc-br/MosViR). It requires R v. 4 and a previous installation of Biostrings (https://bioconductor.org/packages/Biostrings) and mnmer (33) packages. This pipeline aims to classify viral contigs into (i) Mosquito-associated viruses, (ii) Other viruses: (a) MSV and (b) Arboviruses. Employing two sequential classifications, as illustrated in Figure 2, MosViR offers a comprehensive solution for researchers seeking to classify the viral contigs obtained through mosquito sequencing.

The MosViR pipeline requires a fasta-formatted file containing viral contigs, each with a minimum size of 500 bp and varying percentages of non-ACTG bases. The pipeline conducts individual analysis for each contig, starting with quality control. This ensures that the occurrence of these bases does not exceed an acceptable threshold, which is currently set at 2%. The testing was designed to identify a balance between effectively removing low-quality contigs and preserving informative sequences for accurate classification. This stringent quality control step contributes to the overall reliability of the classification process by preventing the inclusion of contigs, which could compromise the accuracy of downstream analyses.

Feature extraction and subset selection are applied to high-quality contigs, with the choice of the classification fragment (500, 1000, 3000, 5000 or 10 000 bp) contingent on the length of the input contig. The subset of contigs is influenced by the proximity of sequence length to the specified fragment.

The MosViR pipeline employs a soft-voting ensemble methodology to conduct classification, with 50 predictive models applied to each fragment length in classification steps one and two. The final predictive models were trained using (1,2)-mers, the best-performing feature capable of capturing intricate sequence discriminatory patterns for all fragment lengths. The classification algorithms associated with (1,2)-mers were selected for their performance and included one of the following: SVM, KNN, LR, RF, LDA or QDA. This approach harnesses the collective predictive power of diverse models to enhance classification accuracy.

After the classification, the pipeline produces an output file with probability scores for each input contig. The scores obtained through soft voting indicate the likelihood of the predictive models being right when assigning a contig to its predicted class. These values range from 0.00 to 1.00 and represent the aggregated predicted probabilities across multiple models. The decision-making process for predicting classes relies on these scores, with a standard threshold set at 0.50. The MosViR pipeline uses the 0.50 threshold as default. However, the user can modify this value to adjust the classification stringency or meet specific requirements.

### Retrieval and pre-processing of metatranscriptomic mosquito data

We assessed the classification capabilities of the MosViR pipeline on real-world inputs by curating data from 24 metatranscriptomic projects documented in previously published literature. All curated projects sequenced mosquito samples using Illumina technology. We retrieved data from the NCBI Sequence Read Archive based on the project ID. Supplementary Table S2 provides the project ID, sample source, total reads, and number of contigs retrieved for each project.

We used the bbduk tool (https://sourceforge.net/projects/bbmap/) to conduct a thorough read quality control analysis involving adapter trimming, low-quality read filtering (Phred quality score > 20 and a minimum read length of 100), and contaminant removal. To eliminate host sequences and endogenous viral elements (EVEs), we used Bowtie2 (36) to map quality reads to mosquito genomes from the genera *Culex* sp. and *Aedes* sp., which are highly prevalent in the curated projects. This approach was previously used to remove EVEs from mosquito metatranscriptomic data (37). Unmapped reads were assembled *de novo* into contigs using SPAdes v. 3.16 (38) with default settings. Contigs over 500 bp and <2% non-ACTG base content were clustered to remove redundancy at 90% nucleotide identity using CD-HIT-EST (http://weizhong-cluster.ucsd.edu/cd-hit/). Supplementary Figure S1 presents a comprehensive diagram describing the analysis conducted to prepare the metatranscriptomic data for classification by the MosViR pipeline.

### Similarity-based search and MosViR classification of previously described viruses from real-world data

Identifying viral contigs involved a similarity-based search against the NCBI viral RefSeq database downloaded in February 2024 using Diamond BLASTX (39) with an e-value threshold of 1e-10. This threshold prioritizes the specificity and minimizes the risk of false positives by reducing the expected number of chance hits in a random alignment. Based on the BLASTX results, we labeled our contigs into three groups: (i) known viruses, (ii) putative novel viruses and (iii) unknown contigs. We labeled contigs with at least 90% amino acid identity to previously described viruses as Known viruses, assigned the label Putative novel viruses to those with <90% identity, and designated contigs without matches as Unknown contigs. Grouping contigs based on their identity levels with known viral genomes was a crucial step in pipeline testing, mirroring the approach employed by previous studies (22).

The contigs labeled as known viruses were used to assess the generalization of our pipeline with real-world data. We confirmed the viral origin of all contigs by conducting a similarity-based search against the NCBI non-redundant database, downloaded in February 2024, using Diamond BLASTX with an e-value threshold of 1e-10 (39). All contigs with hits in other organisms were excluded from subsequent analysis. Using the BLASTX output table, we assigned classes to each contig by selecting the best hits with lower e-values and the highest bit scores. This strategy prioritizes those hits with higher statistical significance and stronger sequence similarity. We cross-referenced the taxonomic information for the best hits with the previously selected viral species during the data collection stages, ensuring precise class assignment. After discarding contigs with the best hits associated with unselected species, we labeled the remaining contigs as Mosquito-associated viruses, Other viruses, Arboviruses, or MSV. The labeled contigs were used to generate feature matrices serving as the testing ground for the MosViR pipeline.

### Identification and MosViR classification of novel mosquito-associated viruses

To assess the capability of our pipeline in predicting novel mosquito-associated viruses, we detected divergent contigs bearing the RNA-dependent RNA polymerase (RdRp) protein from both the Putative novel viruses and the Unknown contigs groups. We used Prodigal (https://github.com/hyattpd/Prodigal) and Prodigal-gv (https://github.com/apcamargo/prodigal-gv) in anonymous mode to predict protein-coding genes, followed by the curation of resulting amino acid sequences to 99% identity using CD-HIT ((http://weizhong-cluster.ucsd.edu/cd-hit/). Functional annotation was performed using the hmmscan (e-value 1e-10) function of HMMER v. 3.4 (http://hmmer.org/). The contigs were scanned against two sources of hidden Markov model profiles (pHMM): RdRp-Scan (23), and Pfam (https://www.ebi.ac.uk/interpro/download/pfam/ downloaded in February 2024). Using multiple sources of pHMM aims to enhance virus detection. However, this approach may also lead to a higher incidence of false positives. To potentially reduce false positive results, we considered sequences as RdRp-bearing only if they had matches from both pHMM sources.

From the RdRp-bearing contigs, we identified previously unreported RdRp proteins by conducting a similarity-based search against the NCBI Non-Redundant database, using Diamond BLASTP (39) in the very sensitive mode (e-value of 1e-10). We considered as novel RdRps the RdRp-bearing contigs matching previously published RdRp proteins and demonstrating less than 90% amino acid identity. This identity criterion has been employed in multiple studies, including those by (14,22). We recovered the nucleotide sequences for all novel RdRp contigs and used them for classification by our pipeline.

### Phylogenetic analysis

Building on the previous functional annotation, we subsetted the novel RdRp contigs according to their homology with specific RdRp domains: RdRP_1 (PF00680), RdRP_2 (PF00978), RdRP_3 (PF00998), RdRP_4 (PF02123), RdRP_5 (PF07925), Birna_RdRp (PF04197), Flavi_NS5 (PF00972), Mitovir_RNA_pol (PF05919), Bunya_RdRp (PF04196), Arena_RNA_pol (PF06317), Mononeg_RNA_pol (PF00946) and Flu_PB1 (PF00602). We subsetted the novel RdRp contigs into 12 viral groups based on their homology to these RdRp domains. The novel RdRp contigs, together with their corresponding homologs and representative species from each order or family retrieved from the RdRp-Scan sequence database, formed the following groups: Picornavirales-like and Nidovirales-like, Tymovirales-like and Hepe-Virga-like, Tombusviridae-like and Nodaviridae-like, Toti-, Luteo-, and Sobemoviridae-like, Reoviridae-like, Birnaviridae-like, Flaviviridae-like, Narnaviridae-like, Bunyavirales-like, Arenaviridae-like, Mononega- and Chuviridae-like, Orthomyxoviridae-like.

For each RdRp domain, we performed amino acid sequence alignment using MAFFT v.7.407 (https://mafft.cbrc.jp/alignment/software/) and the E-INS-I algorithm. We removed ambiguously aligned regions using TrimAl v. 2.0 (https://github.com/inab/trimal), employing an automated trimming heuristic. All multi-sequence alignments were manually checked to ensure the presence of at least two conserved motifs in the RdRp domain. Each multi-sequence alignment was subjected to maximum likelihood phylogenetic analysis as implemented inIQ-TREE v. 2 (https://www.iqtree.org/), with substitution model selection was carried out by the ModelFinder algorithm and branch support assessed by 1 000 bootstrap replicates. The resulting phylogenies were annotated using iTol (https://itol.embl.de).

All data obtained or generated in this study, along with the corresponding scripts, can be openly accessed at https://github.com/labinfo-lncc-br/MosViR_SB and https://github.com/labinfo-lncc-br/MosViR.

## Results

### Overview of training and testing data

The datasets for training and testing the predictive models include 7 808 viral species classified as Other viruses, 92 as Arboviruses, and 298 as MSV (Supplementary Table S3). The Other viruses exhibit a significantly higher number of viral species due to the broader range of hosts, not limited to Diptera insects. Of the 1 076 808 viral nucleotide sequences retrieved from the NCBI RefSeq database, 34.5% (*n* = 371 498) were removed due to redundancy, low quality, partial sequences, or non-conformance with length requirements. The varying number of remaining sequences (8227–694 342) indicates a significant class imbalance.

**Figure 3.**
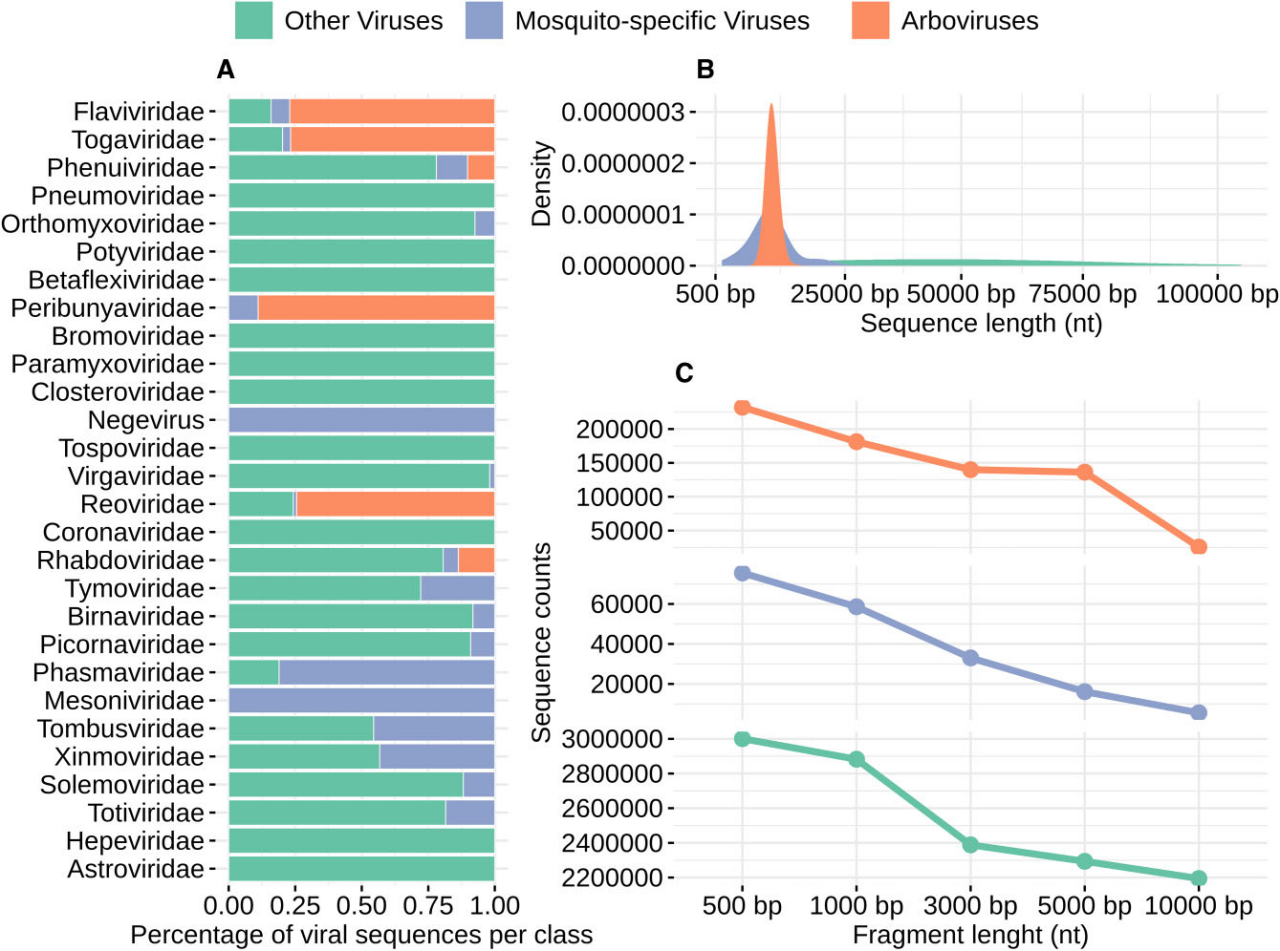
Description of the dataset used in the experiments. (**A**) Percentage of sequences among the classes per viral family. (**B**) Density of sequence sizes per class. (**C**) Number of fragments generated for each fragment length per class.

The taxonomic structure of the remaining sequences retained the pre-selected viral families and species (Supplementary Table S3). The viral families with the highest non redundant sequence count were the Rhabdoviridae (*n* = 202 324, 28.2%), Flaviviridae (*n =* 101 987, 14.2%), Reoviridae (*n =* 101 230, 14.1%), Phenuiviridae (*n =* 75 016, 10.47%), and Togaviridae (*n =* 25 121, 3.5%). These families presented varying percentages of Other viruses, MSV, and Arboviruses (Figure 3A). The remaining sequences used in the dataset present substantial disparity in nucleotide sequence length distributions. Figure 3B shows different density peaks and sequence length variations within a single class and among the classes. Multiple viral families showing divergences in the number of sequences per family (Figure 3A) and high sequence length variability (Figure 3B) contribute to increasing the heterogeneity of the dataset and complexity of the classification process, which is solely dependent on features influenced by hosts. Additionally, the sequence imbalance for Other viruses, MSV, and Arboviruses poses a technical challenge, which is enhanced by the sequence fragmentation process (Figure 3C).

### Model selection and soft voting

By screening feature extraction methods, we identified the optimal feature extraction method for fragment classification at different lengths (500, 1000, 3000, 5000 and 10 000 bp) for two classification steps. Considering the highest mean AUC and specificity as the performance criteria, the (1,2)-mer feature was identified as optimal for discriminating phylogenetically closely related viruses solely by host range (Supplementary Material 1). Across all fragment lengths, this feature extraction method showed mean AUC varying from 0.75 to 0.99 and outperformed all other tested feature extraction methods (Supplementary Table S4). Supplementary Material 1 presents detailed information regarding this analysis.

We individually tested the predictive models built for the (1,2)-mer covering all fragment lengths for (1,2)-mer. These models displayed varying metrics indicating a significant difference in performance across different fragment lengths (Supplementary Table S4). While the AUC values ranged from 0.90 to 0.99 for larger fragment lengths (10 000, 5000 and 3000 bp), the smaller fragment lengths (1000 bp and 500 bp) exhibited a range of 0.75 to 0.89 for the AUC values (Supplementary Table S4), considering the metrics obtained for the test set.

**Figure 4.**
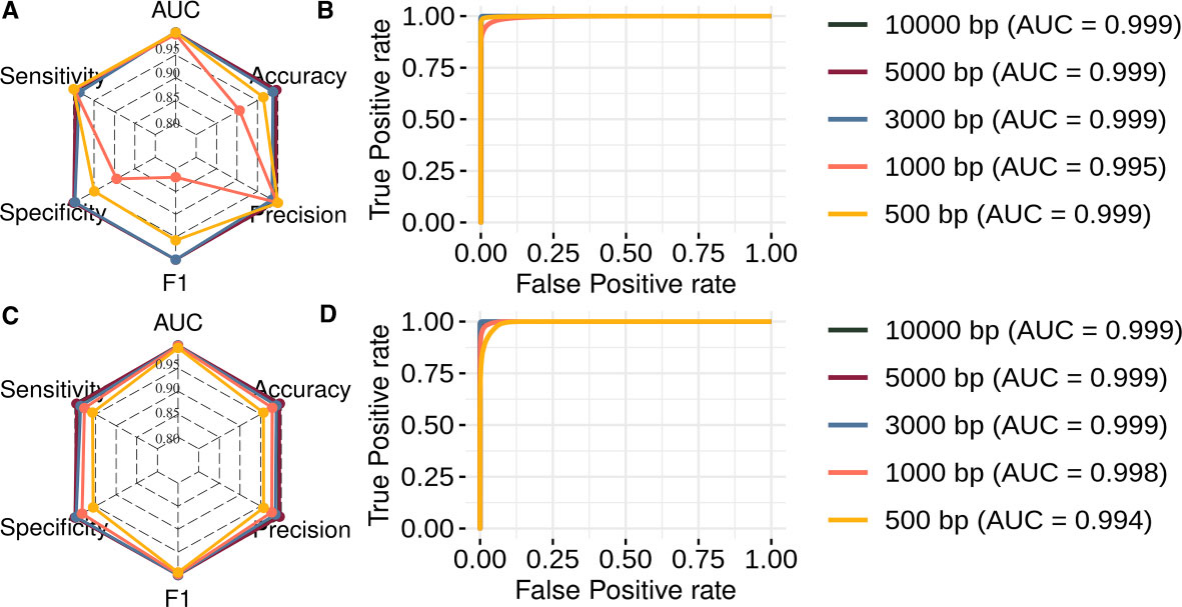
The soft voting performance metrics were obtained by predicting different fragment lengths from testing sets for step 1 (Mosquito-associated viruses versus Other viruses) and step 2 (Arboviruses versus Mosquito-specific viruses). (**A**) Spider plot, and (**B**) ROC curves constructed from the predictions for step 1, (**C**, **D**) metrics for step 2.

Soft voting allowed us to achieve high discriminatory capability with AUCs values close to 0.9 (Figure 4). Compared to the use of single models (Supplementary Table S4), we observed performance improvements for all fragment lengths in both steps, with the most notable improvements for the 1000 and 500 bp. The 1000 bp fragment yielded mean AUC values of 0.89 and 0.79 when using single predictive models (Supplementary Table S4), while soft voting resulted in AUC values of 0.995 and 0.998 for steps 1 and 2, respectively (Figure 4). At 500 bp, the AUC increased from 0.82 and 0.75 with individual models (Supplementary Table S4) to an impressive 0.998 and 0.994 with soft voting for both steps (Figure 4). Considering all fragment lengths, the smallest AUC value observed was 0.994 (500 bp for step 2), with a corresponding specificity of 0.95 and sensitivity of 0.95 (Figure 4). The predictive models mentioned above also generated an AUC of 0.99 when tested only with MSV sequences and an AUC of 0.98 when tested with arboviruses sequences. When all sequences were considered, the AUC value was 0.994 (Figure 4). Similar results were obtained across all fragment lengths, with no significant differences observed when comparing individual classes.

Among the best-performing algorithms used in the soft voting approach, the RF consistently outperformed six others in most resamplings (82% of the cases), followed by the KNN algorithm (10% of the cases) and the SVM algorithm (5% of the cases). These findings underscore the robustness of our models in accurately predicting unseen sequences, even for shorter fragment lengths. These models formed the core of the MosViR pipeline and were later used to predict the presence of mosquito-associated viruses in mosquito metatranscriptomic projects.

### MosViR generalization with metatranscriptomic data from mosquito samples

We collected data from 24 mosquito metatranscriptomic projects to test the MosViR generalizability in real-world classification scenarios. The data encompass a wide range of mosquito species sampled from different countries, with a significant prevalence of *Aedes* sp. and *Culex* sp. We assembled a range of 23 819 contigs for PRJNA924100 to 13 468 621 for PRJNA778885, with a mean of 2 442 696 contigs per project. After applying strict pre-processing, we retained between 44.61% and 89.95% of these contigs, resulting in 1 470 508 contigs across all projects (Supplementary Table S2). The filtered contigs showed significant variability in length, ranging from 500 bp to a maximum of 81 315 bp, with a mean length of 1206 bp. 82% of these contigs ranged from 500 bp to 2000 bp, representing challenges to the classification due to their low informational content compared to larger contigs. By performing similarity-based searches, we labeled 34.7% of these contigs as Known contigs (*n =* 511 138), 23.5% as Putative novel viruses (*n* = 345 569), and 41.8% as Unknown contigs (*n =* 614 672). These percentages fluctuated across individual projects, with detailed project-wise statistics available in Supplementary Table S2.

Out of the 511 138 contigs labeled as Known viruses, 138 007 contigs (27%) were confirmed to belong to previously described viral species through similarity-based searches against the NCBI non-redundant database. The remaining 373 130 contigs (73%) either showed a nucleotide identity smaller than 90% with their best hit or had hits with other organisms and were excluded from further analysis. We used the taxonomic information from each BLASTX best hit to assign classes to the contigs and cross-referenced it against the viral species selected during data collection. Most contigs were associated with Unclassified viruses (taxid: 12 429), with a prevalence of *Riboviria* sp. (taxid: 2 585 031), *Bunyavirales* sp. (taxid: 2 050 579) and *Picornaviridae* sp. (taxid: 1 530 251). These contigs were excluded from the analysis due to a lack of host-range information. Out of 138 007 contigs, 5 618 (4.07%) were assigned to the following classes: Mosquito-associated viruses (*n =* 708), Other viruses (*n =* 873), MSV (*n =* 693) or Arboviruses (*n =* 15). These contigs may contain incomplete sequences encoding various viral proteins with a range of identities from 90% to 99% with their best match and varied in length from 500 bp to 29 677 bp, with a mean length of 1790 bp. The stringent class assignment led to curating a refined database containing 5618 contigs capable of accurately testing the MosViR performance in classifying previously described viruses.

**Figure 5.**
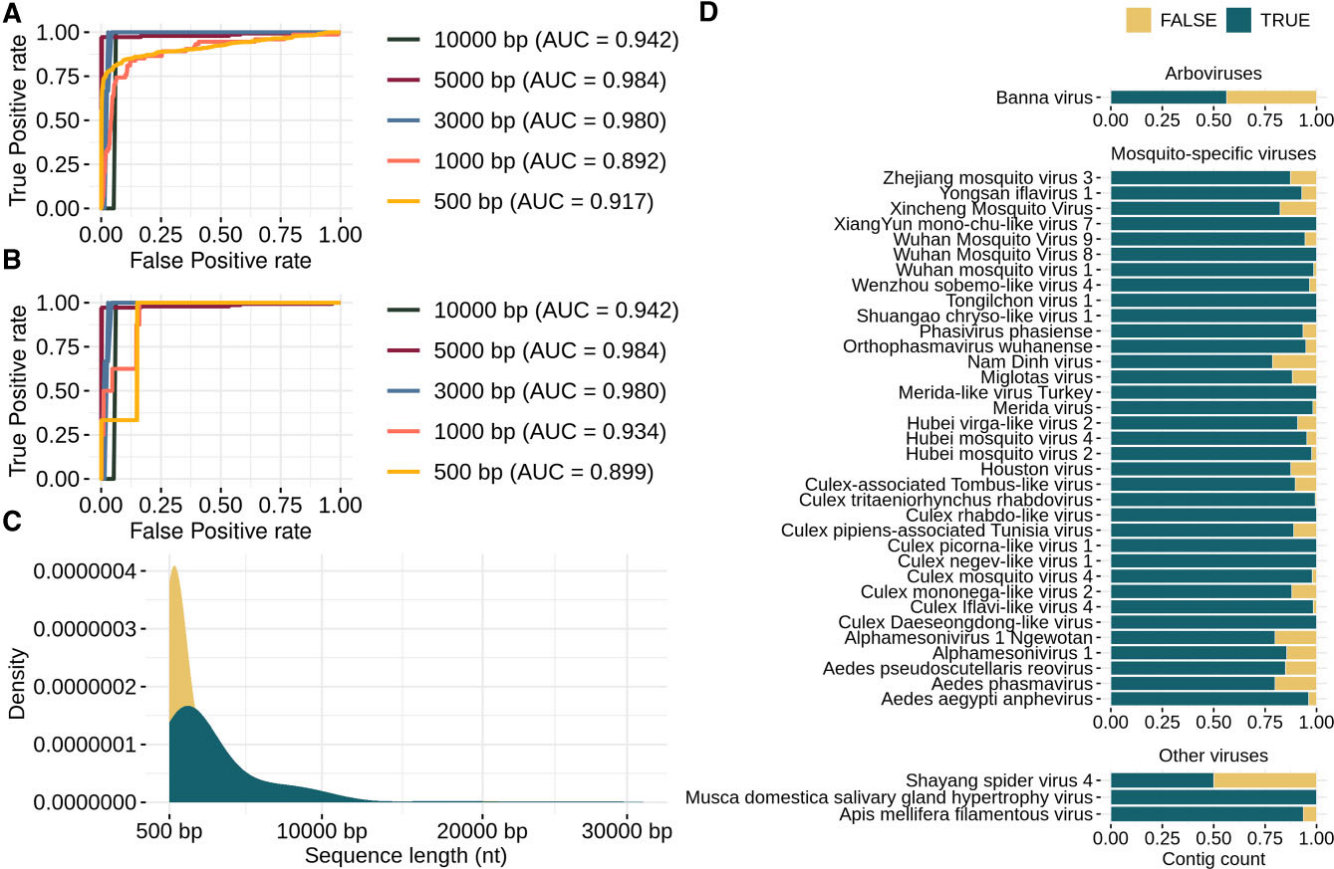
MosViR prediction for the 5618 known contigs. (**A**, **B**) showcase the ROC curves and corresponding AUC values for the first and second steps of prediction, respectively. (**C**) The density of nucleotide length for contigs classified either as True or False. (**D**) Top 30 species with most contig counts in the dataset. The species were ordered from highest to lowest contig count within a class. The bar plot shows the percentage of True predictions (True Positives and True Negatives) and False predictions (False Positives and False Negatives).

We assessed the MosViR performance on Known virus contigs, splitting them into five testing sets based on contig length for independent classification. As a result of MosViR’s soft voting approach, ROC curves and AUC values range from 0.89 to 0.98 (Figure 5A and B), indicating robust performance in classifying real-world data across diverse contig lengths. All testing sets showed contigs for positive and negative classes, varying from 80 (for the 5000 bp in step 2) to 1669 contigs (for the 1000 bp in step 1). Further, probability scores for positive classes were analyzed for TP, TN, FP and FN outcomes in both steps. Our classifications showcased higher scores (median of 0.8) associated with TP and lower scores (median of 0.07) for TN. Intermediate scores (medians closest to 0.5) were associated with FP and FN outcomes.

MSV presented species with the highest contig counts in the Known virus dataset. For instance, *Culex tritaeniorhynchus rhabdovirus* (taxid: 936 308) presented 1662 contigs, followed by *Hubei mosquito virus 2* (taxid: 1 922 926) with 1 044 contigs. The species with the highest contig count for the Other viruses was *Apis mellifera filamentous virus* (taxid: 1 100 043) with 61 contigs, and for the Arboviruses was *Banna virus* (taxid: 77 763) with 32 contigs. We identified contigs of different lengths and diverse classification outcomes within the same species (Figure 5D). The 28 contigs associated with the *Shayang spider virus 4* (taxid: 1 746 062, 28 contigs) showed a true outcome rate of 50%. Similarly, the true outcome rate for the *Banna virus* (taxid: 77 763, 32 contigs) was 62%. These findings may be related to the association between shorter contig lengths (500 and 1000 bp) with higher false outcome rates (Figure 5C).

The Putative novel contigs (*n* = 345 569) and the Unknown contigs (*n =* 614 672) were used to identify 8679 RdRp-bearing contigs through functional annotation. The subsequent similarity-based search refined the contigs to 1560 novel RdRp contigs, each presenting <90% amino acid identity against the NCBI non-redundant database. Given the novelty of the viruses being tested, we considered the probability score for the predicted class as the main parameter during the decision-making process of class prediction. The novel RdRp contig count for the Other viruses varied from 298 to 14, considering score thresholds from 0.5 to 0.9 in order of stringency. The Arboviruses yielded fewer classified contigs, ranging from 58 to 3 for the less stringent threshold to the most stringent score threshold, respectively. In contrast, the MSV had the highest contig count, ranging from 1204 for the 0.5 threshold to 420 for the 0.9 threshold (Supplementary Figure S2A). Interestingly, 120 MSVs were identified with a probability score of 1.

### Phylogenetic analysis of newly identified mosquito-associated viruses

Based on the previous classification of known contigs (Figure 5), we selected a probability score threshold of 0.70 to achieve a balance between minimizing false outcomes and preserving true outcomes. We identified 641 novel RdRp contigs that were classified as Other viruses (*n* = 36), MSV (*n* = 597) and Arboviruses (*n* = 8) with divergent RdRp domains (Supplementary Figure S2B and Supplementary Table S5). These contigs presented varying lengths ranging from 500 bp to 12 000 bp (Supplementary Figure S2C), with a mean of 912 bp and 40% to 89.9% of amino acid identity with their best hit against the NCBI non-redundant database. We inferred the evolutionary relationships of novel mosquito-associated RdRp contigs (MSV and Arboviruses) by constructing phylogenetic trees for each RdRp domain and their representative sequences from previously described viruses. Noteworthy, the RdRp_5 and Arena_RNA_pol domains had no hits during functional annotation, and as a result, corresponding phylogenetic trees were not constructed (Supplementary Table S5).

Considering all trees inferred for the 605 novel mosquito-associated RdRps, most contigs (*n* = 498, 82.3%) clustered among themselves and were not associated to previously reported clades, indicating significant evolutionary divergence of the novel RdRps (Supplementary Material 2). Of the remaining novel mosquito-associated RdRps, 14% (*n* = 85) presented a host-range agreement with closely related viruses belonging to MSV or Arboviruses clades. Other 3.6% (*n* = 22) novel RdRp classified as MSV clustered within plant and fungal pathogens from the Bromoviridae and Alphaflexiviridae.

Multiple novel mosquito-associated RdRps are closely related to other novel RdRps sequenced by multiple metatranscriptomic projects. This finding suggests that these newly identified viruses may be circulating in multispecies mosquito populations worldwide. These novel mosquito-associated RdRps had a high prevalence of best hits to Unclassified viruses, especially the *Riboviria* sp., and environmental samples, as well as multiple viruses for which the taxonomic classification is not determined, such as *Bunya-like virus*, *Virga-like*, *Negev-like virus*, *Gouko-like* and *Tombus-like* viruses.

The novel dsRNA mosquito-associated clustered within the Totiviridae (*n* = 51), Sobemoviridae (*n* = 30), Luteoviridae (*n* = 8), Birnaviridae (*n* = 1) clades in the phylogenetic trees built for the BirnaRdRp and RdRp_4 domains. The probability scores ranged from 0.1 to 0.6, with a high prevalence of *Khabarov virus*, *Kisumo mosquito virus* and *Aedes aegypti toti*-like virus as the best hits.

**Figure 6.**
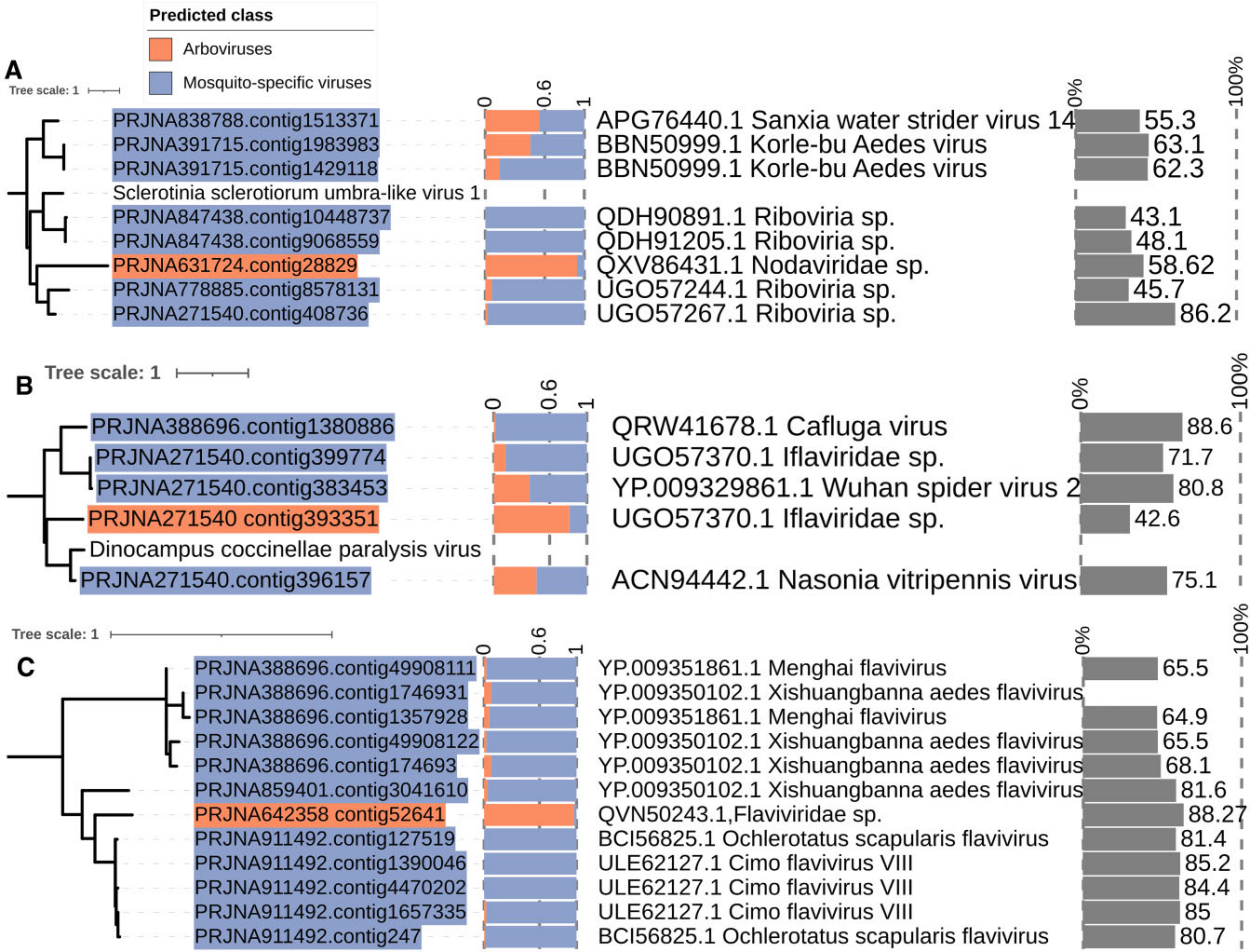
Phylogenetic relationships between novel RdRp contigs classified as Arboviruses within the positive sense RdRp domains RdRp_3, RdRp_1 and FlaviNS5. (**A**) Nodavirida family (RdRp_3 domain), (**B**) Iflaviviridae family (RdRp_1 domain), (**C**) Flaviviridae family (FlaviNS5 domain). Novel RdRp contigs predicted as novel Arboviruses are highlighted in orange, while novel Mosquito-specific viruses are represented in purple. The bar plot shows the probability score for the positive class per contig, emphasizing the score threshold set at 0.6. Additionally, the tree plot displays taxonomic information and identity scores based on amino-acid similarity-based searches against the NCBI non-redundant database.

The novel ssRNA+ (Baltimore class IV) mosquito-associated viruses clustered mainly within the clades of Picornaviridae (*n* = 109), Iflaviviridae (*n* = 41), Virgaviridae (*n* = 26, 44.8%), Flavivirus genera (*n =* 62), and Negevirus taxon (*n =* 23, 39%). We classified three novel RdRp as Arboviruses: one for the Nodaviridae family (RdRp_3 domain), one for the Flaviriridae (FlaviNS5), and Iflaviriridae families (RdRp_1 domain). The novel arbovirus identified for the RdRp_3 domain presented a probability score of 0.95, length of 2226 bp, and clustered with novel RdRp for MSV that had best hits with Unclassified RNA viruses (Figure 6A). The novel Arboviruses identified for the Flaviviridae family clustered within the Flavivirus genera, exhibiting close relationships to well-described MSV. This 511 bp virus sequence presented a probability score of 0.97 (the highest probability score identified for novel Arboviruses). For the Iflaviviridae family, the novel Arboviruses presented a length of 910 bp and a probability score of 0.81.

**Figure 7.**
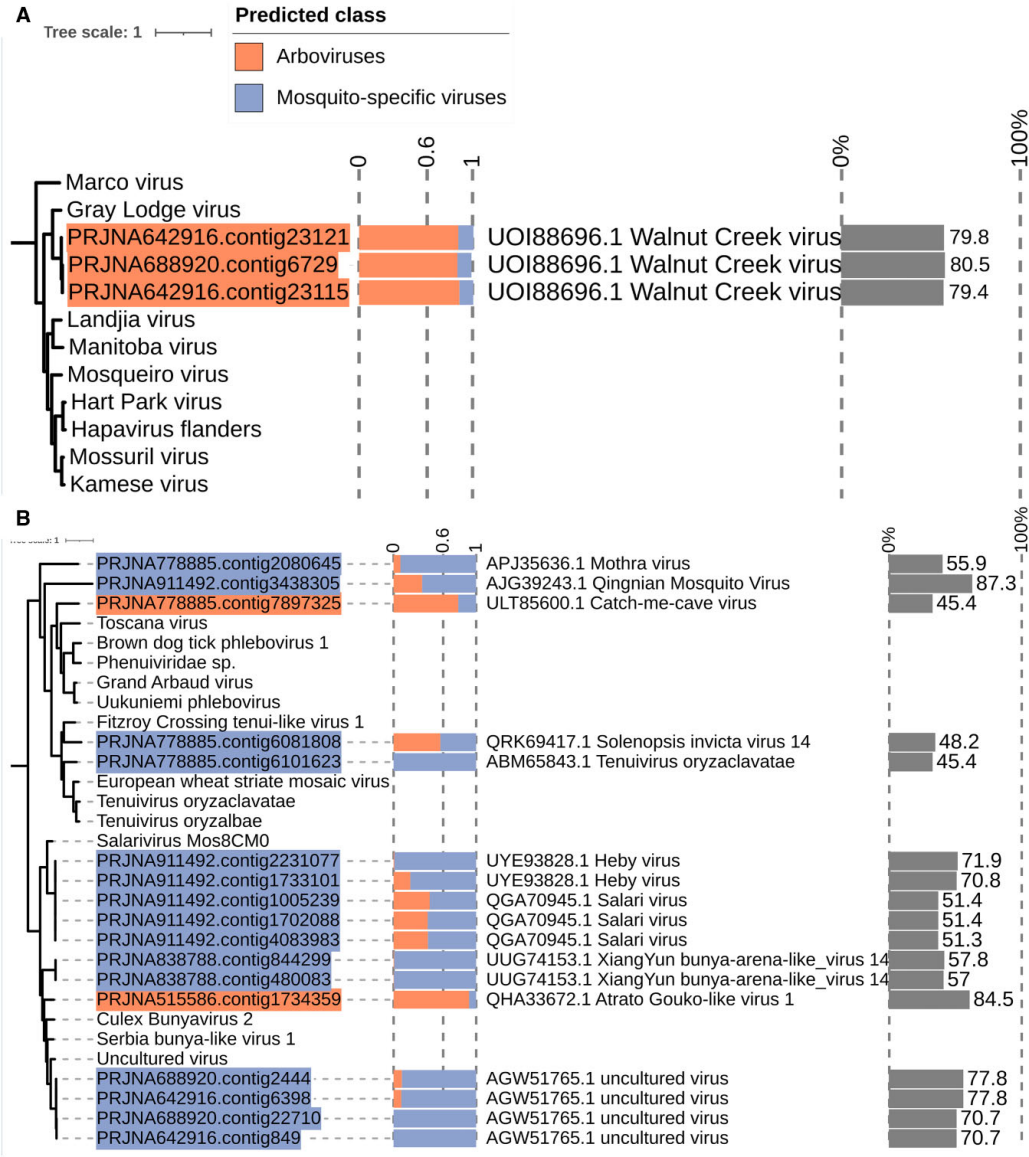
The maximum likelihood phylogenetic trees show the phylogenetic relationships among the novel RdRp classified as Arboviruses and their closest representative sequences from the Mononega_pol_RNA and BunyaRdRp domains. (**A**) From the Mononega_pol_RNA, the Rhabdoviridae family presented three novel RdRp classified as Arboviruses. These contigs clustered with members of the Hapavirus genera. (**B**) For the BunyaRdRp domain, the Phlebovirus genera from the Phenuiviridae presented a novel RdRp classified as Arboviruses and a novel RdRp clustered with Unclassified viruses.

For the novel ssRNA- viruses (Baltimore class V) mosquito-associated viruses, we identified clusters within the Rhabdoviridae (*n* = 56), Xinmoviridae (*n* = 18), Chuviridae (*n* = 16), Orthomyxaviridae (*n* = 13), Phenuiviridae (*n* = 6), Peribunyaviridae (*n* = 2) and Phasmaviridae (*n* = 1). A considerable amount clustered with Unclassified viruses (*n* = 17). For the Mononeg_RNA_pol domain, we identified three novel RdRp classified as Arboviruses in the Rhabdoviridae family. These RdRps clustered with the Hapavirus genera, presenting close evolutionary relationships to the previously identified arboviruses *Gray Lodge virus*, *Mosqueiro virus*, *Landjia virus*, *Hart Park virus* and *Flanders virus*. These RdRp ranged from 611 to 811 bp, with probability scores from 0.82 to 0.88, and best hit the *Walnut Creek virus* with amino acid identities from 79.4 to 80.5% (Figure 7A). In the BunyaRdRp domain, we identified two novel RdRp classified as Arboviruses. The first RdRp clustered with Phenuiviridae within the clade for the genera phlebovirus that harbors multiple Arboviruses, including the closely related *Toscana virus* (Figure 7B). This novel Arboviruses presented a significant nucleotide length of 6452 and a probability score of 0.78. The second RdRp classified as Arboviruses for the BunyaRdRp domain clustered with Uncultured virus obtained through mosquito sequencing and *XiangYun Bunya-arena-like virus*. This RdRp presented a length of 698 bp and a probability score of 0.91 (Figure 7B). Interestingly, the BunyaRdRp domain presented five novel RdRps classified as MSV with scores ranging from 0.56 to 0.68 that clustered with Peribunyaviridae, Phenuiviridae and Unclassified virus clades.

No novel mosquito-associated RdRp clustered within families with no documented associations to Diptera insects, such as Coronaviridae, Filoviridae, Paramyxoviridae, Bornaviridae, and Pneumoviridae (Supplementary Material 2).

## Discussion

We developed the MosViR pipeline to uncover novel MSVs and Arbovirus sequences as short as 500 bp, which were previously overlooked by traditional homology-based methods. MosViR is based on the hypothesis that viral genetic composition reflects distinct evolutionary pressures shaped by a shared host environment (26,27). Similarly to previous studies, the significant sequence imbalance in databases potentially biases analyzes toward the most represented viral taxa, impacting the universality of identified features (27). Although MosViR predictions are highly accurate, the discriminatory patterns used to classify mosquito-associated viruses remain to be elucidated in a higher depth.

The predictive models in MosViR demonstrate high discriminatory capabilities, effectively classifying shorter fragment lengths ranging from 500 bp to 1000 bp with low FP rates. This performance surpasses the well-known challenge of classifying short, non specific, and low informational content sequences (32,33). Notably, our approach of combining multiple predictive models, rather than relying on single models, further enhanced accuracy for shorter fragments, as indicated by previous studies (34,35). In our classification scenarios, the FP results (false mosquito-associated viruses and false arboviruses) pose a greater concern than those of FN. We increase the probability threshold from the default 0.50 to 0.70 to reduce their occurrence. This adjustment led to highly accurate positive classifications while inflating the FN results. By adjusting this parameter, users can optimize classification accuracy and find the right balance between false outcomes according to their research goals.

The MosViR pipeline offers broader applicability for classifying mosquito-associated viral sequences. The alignment-free complex discriminatory patterns that MosViR uses to infer host-range associations can be explored by classifying previously described viruses from different taxonomic organizations and comparing the frequency for specific (1,2)-mers. Previously described mosquito-associated viruses can be classified to monitor possible host shifts from MSVs to Arboviruses (9,10). The MosViR pipeline can also provide new information about unknown viral sequences and classify novel viruses. The pipeline receives viral contigs as input, which are obtained by assembling reads from metatranscriptomic sequencing of environmental samples or tissues from the vertebrate host or mosquito vectors. However, identifying viral sequences does not inherently indicate active replication in potential hosts. In virome studies, even the sequences commonly detected in mosquitoes will need further wet lab experiments to verify their host range and potential pathogenicity (5). Therefore, MosViR output can be used to select candidates for experimental analysis, enabling the discovery of potential viral pathogens and MSVs that can act as biocontrol agents.

Our accurate classification of phylogenetically closely related viruses highlights the ability to overcome critical challenges in viral classification, such as phylogenetically structured noise and taxonomy reflection. These well-known challenges occur when a predictive model learns patterns based on the shared evolutionary history of viruses rather than specific traits. This leads to inflated performance metrics that do not generalize well to novel cases (28). Our findings suggest that the MosViR pipeline offers a potential solution for these challenges when classifying mosquito-associated viruses from sequences as short as 500 bp.

Here, MosViR pipeline classified 605 novel mosquito-associated viruses identified by mining metatranscriptomic dark matter. Given the lack of metatranscriptomic sequencing of vertebrate host tissues, we focused on analyzing mosquito viromes. Our methodology involved retrieving functional RdRp domains to perform classifications and construct phylogenetic trees, which is in line with multiple previous studies that described viral sequences from dark matter (17,22). However, this approach significantly limited the classification of highly divergent RdRp or sequences containing other viral genes as MosViR is not constrained by RdRp-bearing sequences.

The newly identified mosquito-associated viruses showed 40–89% amino acid identity to their most closely related viruses, indicating virus diversity at species, genera, and family levels (13–15). This diversity is evidenced by most of these viruses (82.3%) forming distinct clades, restricting their classification and assessment of host range using homology-based methods. The distinct clades are predominantly formed by novel mosquito-associated sequences retrieved from independent metatranscriptomic projects encompassing multiple mosquito species collected from locations worldwide from 2018 to 2024. This finding suggests that these novel mosquito-associated viruses may constitute a previously undescribed part of the mosquito core virome, and could potentially serve as biocontrol agents (7,11,12). Further investigations are essential to correlate these novel mosquito-associated viruses with species-specific core viromes, elucidate their life cycle, and assess their potential to reduce vector competence in transmitting medically significant arboviruses. Overall, these findings highlight the ability of MosViR to comprehensively characterize the mosquito virome, providing unique insights into the diversity of RNA viruses present in mosquito populations.

Our findings highlight using the MosViR pipeline to expand the mosquito-associated virosphere, even when encompassing novel or unexpected taxa. The families with the highest count of novel mosquito-associated viruses were Picornaviridae, Flaviviridae, Rhabdoviridae, Xinmoviridae, Virgaviridae and Totiviridae. Additionally, we found several novel mosquito-associated viruses grouped within clades for the unclassified taxa Chuvirus (14) and Negevírus (40), as well as clades for families recently known to infect mosquitoes, such as the Iflaviridae, Dicistroviridae, Totiviridae, Mitoviridae, Sobemoviridae, Luteoviridae, and Birnaviridae (13–15). These findings echo previous reports showing the identification of mosquito-associated viruses in novel clades (13–15,17,40).

The MosViR pipeline classified eight putative novel arboviruses with a high predicted probability for correct class assignment (ranging from 0.78 to 0.95) by repurposing unclassified sequences. The putative novel arboviruses clustered with Rhabdoviridae, Flaviviridae, Nodaviridae, Phenuiviridae, Iflaviviridae, and Bunyavirales clades. Except for the Iflaviviridae family, all clades are well-known to harbor multiple arboviruses causing high-burden disease in humans (4,5). Although the putative novel Arboviruses encompass incomplete genomes, the high predicted probability score strongly suggests a host-range agreement with previously described arboviruses. Considering homology-based methods are often limited to predict potential novel arboviruses with close evolutionary relationships with well-described pathogens (10,13). Our findings highlight the MosViR potential to overcome these limitations and perform wider genomic surveillance to identify complex discriminatory patterns suggestive of the arbovirus dual-host life cycle in unclassified sequences.

The phylogenetic analyses revealed no clustering between the sequences classified as mosquito-associated viruses by MosViR and viral families without clear associations to arthropods. Mosquito viromes are expected to contain contaminant sequences, especially viruses infecting mosquito microbiota or obtained from undigested food. However, a high false positive rate could influence the classification of viruses, showing considerable phylogenetic divergence from known mosquito-infecting viruses (5,13). This finding emphasizes the accurate prediction of unclassified viruses and reinforces the robust discrimination between genuine mosquito-associated viruses and potential false positives.

Given the substantial disease burden caused by arboviruses and the potential emergence of novel pathogens transmitted by mosquitoes, heightened mosquito virome surveillance is imperative for early pathogen detection and characterization of the mosquito core virome. In light of these concerns, developing and utilizing computational tools such as MosViR have become increasingly crucial. MosViR offers the capacity to automate the process of assessing the public health risk associated with novel or unclassified mosquito-associated viral sequences. By leveraging MosViR’s capabilities, metatranscriptomic arbovirus surveillance efforts can be significantly enhanced, providing valuable insights into viral diversity and potential threats. As such, future metatranscriptomic studies stand to benefit substantially from the integration of MosViR into their workflows. Additionally, by exploring the intricate discriminatory patterns of Arboviruses and Mosquito-specific viruses, researchers can uncover specific patterns related to host restriction, adaptation to the vertebrate host immune response, and the potential for host shifts from newly identified Mosquito-associated viruses. As our contributions potentially facilitate transformative changes and enhance knowledge in the field of virology, MosViR represents a pivotal tool in the ongoing efforts to combat arboviral diseases and mitigate their impact on public health.

## Conclusion

We developed the MosViR pipeline to uncover mosquito-associated viruses often overlooked by current homology-based methods. MosViR is released as an R package, offering an easy way to accurately identify complex discriminatory patterns in viral contigs and predict their potential host range. Our findings highlight the MosViR pipeline's transformative impact on future mosquito virome studies, enhancing our ability to capture the diverse genomic landscape of mosquito-associated viruses and repurpose large portions of unclassified sequences. We encourage researchers to use MosViR to explore the virome dark matter, identify putative novel mosquito-associated viruses, perform surveillance for host shifts, and select ideal viral candidates for onerous isolation and inoculation experiments.

## Supplementary Material

lqae077_Supplemental_Files

## Data Availability

The MosViR pipeline is available at https://zenodo.org/records/10950999 and https://github.com/labinfo-lncc-br/MosViR. The data underlying this article are available at https://zenodo.org/records/10975801, https://zenodo.org/records/10975789 and https://github.com/labinfo-lncc-br/MosViR_SB.
