# Supplementary material for: Predicting novel mosquito-associated viruses from metatranscriptomic dark matter
Source: NAR Genom Bioinform. 2024 Jul 2;6(3):lqae077. doi: 10.1093/nargab/lqae077 (PMC11217672; doi:10.1093/nargab/lqae077)
Supplement: lqae077_Supplemental_Files [file lqae077_supplemental_files.zip › Captions.docx]

**Captions for Andrade et al 2024**

# **Supplementary Figure 1.** A step-by-step diagram of the comprehensive similarity-based methodology employed for preparing previously published metatranscriptomic data to test the MosViR pipeline.

# **Supplementary Figure 2.** Classification of novel RdRp contigs at 0.5 probability score. All contigs present at least two RdRp motifs and less than 90% identity against their best hit with the Non-redundant database from the National Center for Biotechnology Information (NCBI). A) Count of novel RdRp contigs classified as Other viruses (highlighted green), Mosquito-specific viruses (highlighted purple), and Arboviruses (highlighted orange) considering three scores threshold (0.5, 0.6, and 0.9) B) Percentage distribution of novel RdRp contigs for RdRp domain, C) Density of sequence length distribution for classified novel RdRp contigs.

**Supplementary Table 3.** Overview of Viral Diversity and Sequence Retrieval Across Three Classes.

**Supplementary Table 4.** Feature extraction method selected for each fragment length from both steps. The predictive models exhibited varying percentages of True Negatives (TN), True Positives (TP), False Negatives (FN), and False Positives (FP), along with Area Under the ROC curve (AUC), Specificity, Sensitivity, and Precision.

**Supplementary Table 5.** Functional annotation for the 641 novel RdRp contigs identified in this study, using the probability score threshold 0.7. The contigs vary in functional annotation of RdRp domains, the number of novel RdRp contigs per domain, and the distribution of contigs among different classes.

# **Supplementary Material 1. Selection of feature extraction methods.**

**Supplementary Material 2. Phylogenetic analysis of novel RdRps for each RdRp domain.**
