# Supplementary material for: Predicting novel mosquito-associated viruses from metatranscriptomic dark matter
Source: NAR Genom Bioinform. 2024 Jul 2;6(3):lqae077. doi: 10.1093/nargab/lqae077 (PMC11217672; doi:10.1093/nargab/lqae077)
Supplement: lqae077_Supplemental_Files [file lqae077_supplemental_files.zip › SM1_Andrade_et_al.pdf]

Supplementary Material 1. Selection of feature extraction methods.

A feature selection screening was performed using five feature extraction methods: K-mers, (m,n)-mers [1], Frequency Chaos Game Representation [2], Feature Frequency Profiles [3], and Composition vector [4]. As a result of their ability to successfully classify highly divergent viral sequences based on short k-values, these feature extraction methods were considered for this analysis [1-5].

To manage computational costs, we extracted features with k ranging from two to four for each fragment length (500 bp, 1,000 bp, 3,000 bp, 5,000 bp, and 10,000 bp) considering only the second classification step (Mosquito-specific viruses vs Arboviruses) for the 500 bp fragment length (Table 1). The extraction of k-mers and (m,n)-mers was performed using the mnmer R package [1], while the Alfree software [6] was used for extracting all other features using default parameters. These feature matrices can seamlessly replace one another without requiring additional modifications during the subsequent stages of analysis.

Table 1. Number of feature matrices produced for each feature extraction method, considering the classification of 500 bp fragment length for step 2.

Feature extraction method	K-values	N. of feature matrices
K-mer	2,3, and 4	3
(m,n)-mer	2,3, and 4	6
Frequency Chaos Game Representation	2,3, and 4	3
Feature Frequency Profiles	2,3, and 4	3
Composition vector	3 and 4	2
**Total**	-	17

Each feature matrix was divided into training (70%) and testing (30%). To handle the imbalanced nature of our datasets, we performed 50 random resamplings of the feature matrices. We trained the random forest algorithm and tested the models using the Caret package

(topepo.github.io/caret/) at default parameters for each resampling. Considering the median AUC values, we identified the k-mers and (m,n)-mers as the best-performing feature methods for classification step 2 - fragment length 500 bp (Figure 1).

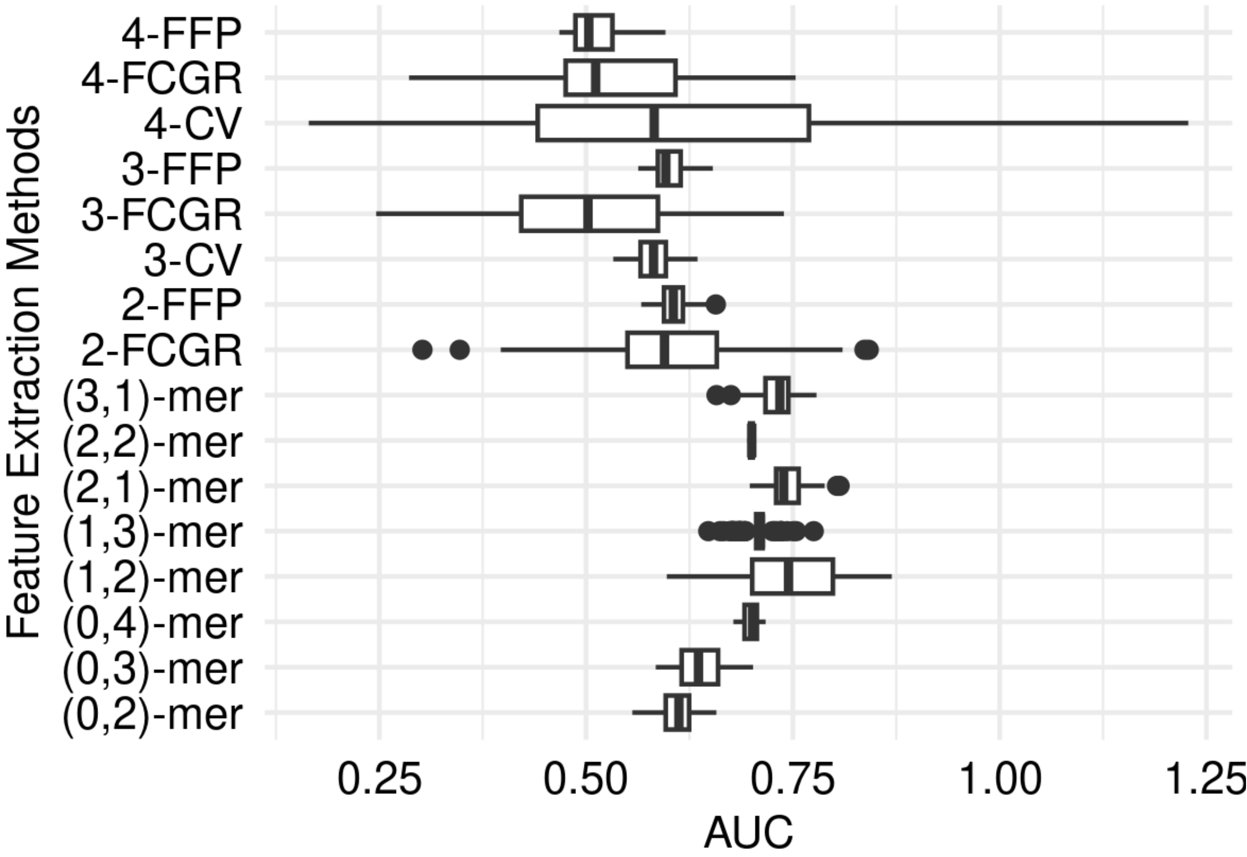

Figure 1. Boxplot for the AUC values retrieved through 50 random resampling for each feature extraction method.

We re-analyzed the k-mers and (m,n)-mers in the context of classification steps one and two and k ranging from two to four, producing eight feature matrices for each fragment length 500 bp, 1,000 bp, 3,000 bp, 5,000 bp, and 10,000 bp. Figure 2 shows mean AUC values obtained from testing the predictive models using soft voting approaches. The (1,2)-mer was identified as the best-performing feature extraction method to use to build our predictive models.

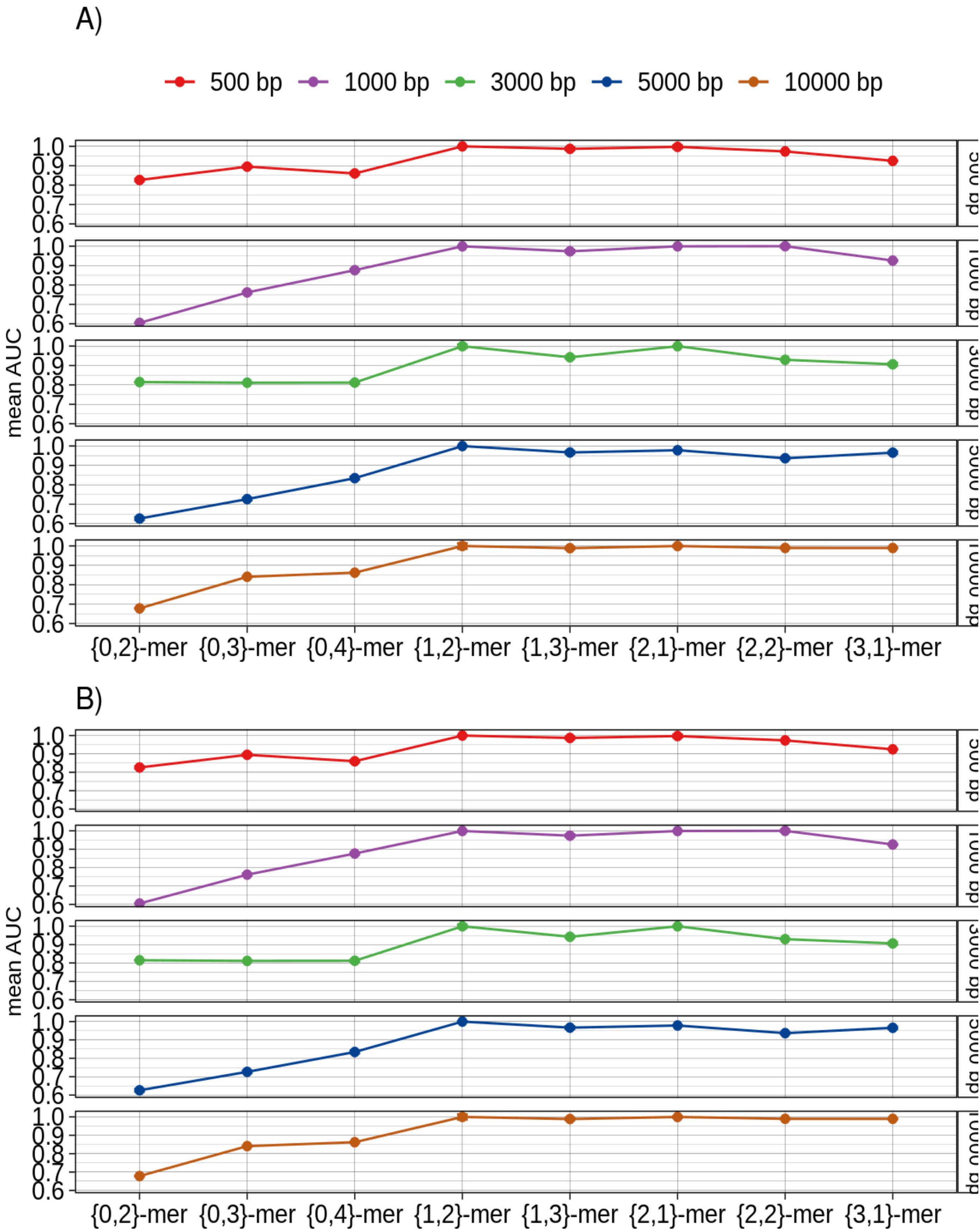
A)
500 bp
1000 bp
3000 bp
5000 bp
10000 bp
mean AUC
1.0
0.9
0.8
0.7
0.6
{0,2}-mer
{0,3}-mer
{0,4}-mer
{1,2}-mer
{1,3}-mer
{2,1}-mer
{2,2}-mer
{3,1}-mer
B)
mean AUC
1.0
0.9
0.8
0.7
0.6
{0,2}-mer
{0,3}-mer
{0,4}-mer
{1,2}-mer
{1,3}-mer
{2,1}-mer
{2,2}-mer
{3,1}-mer

Figure 2. A comparison of the classification performances of (m,n)-mer and k-mer after 50 random resamplings. **A)** Performance for step 1 (Mosquito-associated vs Other viruses), and **B)** Performance for step 2 (Arboviruses vs Mosquito-specific viruses). The figure shows the mean and confidence interval for AUC values. In most cases, the confidence intervals are barely visible on the current scale.

The input data, output metrics, and customized scripts used in our analysis are available at github.com/labinfo-lncc/MosViR.

References

1. Andrade, A. A. S; Grivet, M; Brustolini, O; Vasconcelos, A. T. R. (*m*, *n*)-mer—a simple statistical feature for sequence classification, *Bioinformatics Advances*, Volume 3, Issue 1, 2023, vbad088, https://doi.org/10.1093/bioadv/vbad088

2. Avila Cartes, J., Anand, S., Ciccolella, S., Bonizzoni, P. & Della Vedova, G. Accurate and fast clade assignment via deep learning and frequency chaos game representation. *Gigascience* 12, giac119 (2022).

3. Randhawa, G. S., Hill, K. A. & Kari, L. ML-DSP: Machine Learning with Digital Signal Processing for ultrafast, accurate, and scalable genome classification at all taxonomic levels. *BMC Genomics* 20, 1–21 (2019).

4. Feng, S. et al. A general and transferable deep learning framework for predicting phase formation in materials. npj *Computational Materials* 7, 1–10 (2021).

5. Ren, J., Ahlgren, N. A., Lu, Y. Y., Fuhrman, J. A. & Sun, F. VirFinder: a novel k-mer based tool for identifying viral sequences from assembled metagenomic data. *Microbiome* 5, 69 (2017).

6. Zielezinski, A., Vinga, S., Almeida, J. & Karlowski, W. M. Alignment-free sequence comparison: benefits, applications, and tools. *Genome Biol.* 18, 1–17 (2017).
