# Supplementary material for: Predicting novel mosquito-associated viruses from metatranscriptomic dark matter
Source: NAR Genom Bioinform. 2024 Jul 2;6(3):lqae077. doi: 10.1093/nargab/lqae077 (PMC11217672; doi:10.1093/nargab/lqae077)
Supplement: lqae077_Supplemental_Files [file lqae077_supplemental_files.zip › SM2_Andrade_et_al.pdf]

Supplementary Material 2. Phylogenetic analysis of novel RdRps for each RdRp domain.

We generated ten phylogenetic trees, each corresponding to a distinct RdRp domain with matches for novel RdRp contigs. This Supplementary Material provides an in-depth overview of the phylogenetic analysis. To build the trees, we used the respective novel RdRp contig along with representative sequences from viral species that share taxonomic classification consistent with the RdRp domain. These sequences encompass complete protein amino acids, have undergone prior curation, and are linked to well-described viral entities. All representative sequences were retrieved from the RdRp-Scan database.

1 RdRp domain: BirnaRdRp (PF04197)

For the BirnaRdRp domain, we selected 8 sequences from the Birnaviridae viral family to use as reference sequences. The sequences represented the genera: Aquabirnavirus, Avibirnavirus, Blosnavirus, Dronavirus, Entomobirnavirus, and Telnavirus. This family showed a single match with Mosquito-specific viruses (score: 0.81) that presents as best hit the Rotifer birnavirus at 55.6% of identity.

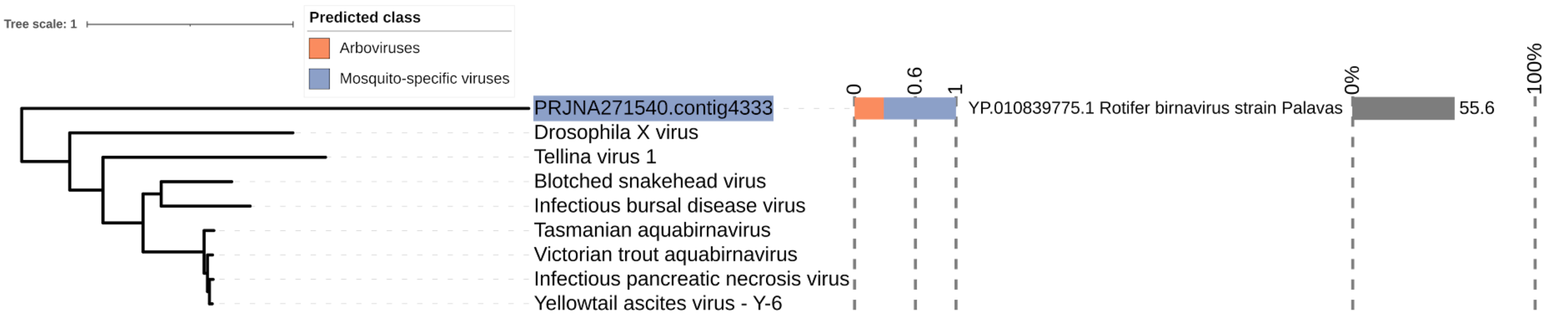

Figure 1. Phylogenetic relationships within the Birnaviridae family (RpRp domain: BirnaRdRp - PF04197). Novel RdRp contigs predicted as novel Arboviruses are highlighted in orange, while novel Mosquito-specific viruses are represented in purple. The bar plot shows the probability score for the positive class per contig, emphasizing the score threshold set at 0.7. Additionally, the tree plot displays taxonomic information and identity scores based on the best hits against the NCBI non-redundant database.

2. RdRp domain: RdRP_4 (PF02123)

For the RdRp_4 domain, we retrieved 92 representative sequences for the families Totiviridae, Luteoviridae, and Sobemoviridae. This domain matched with 89 novel RdRp contigs. All clades without a novelRdRp contig collapsed in the tree.

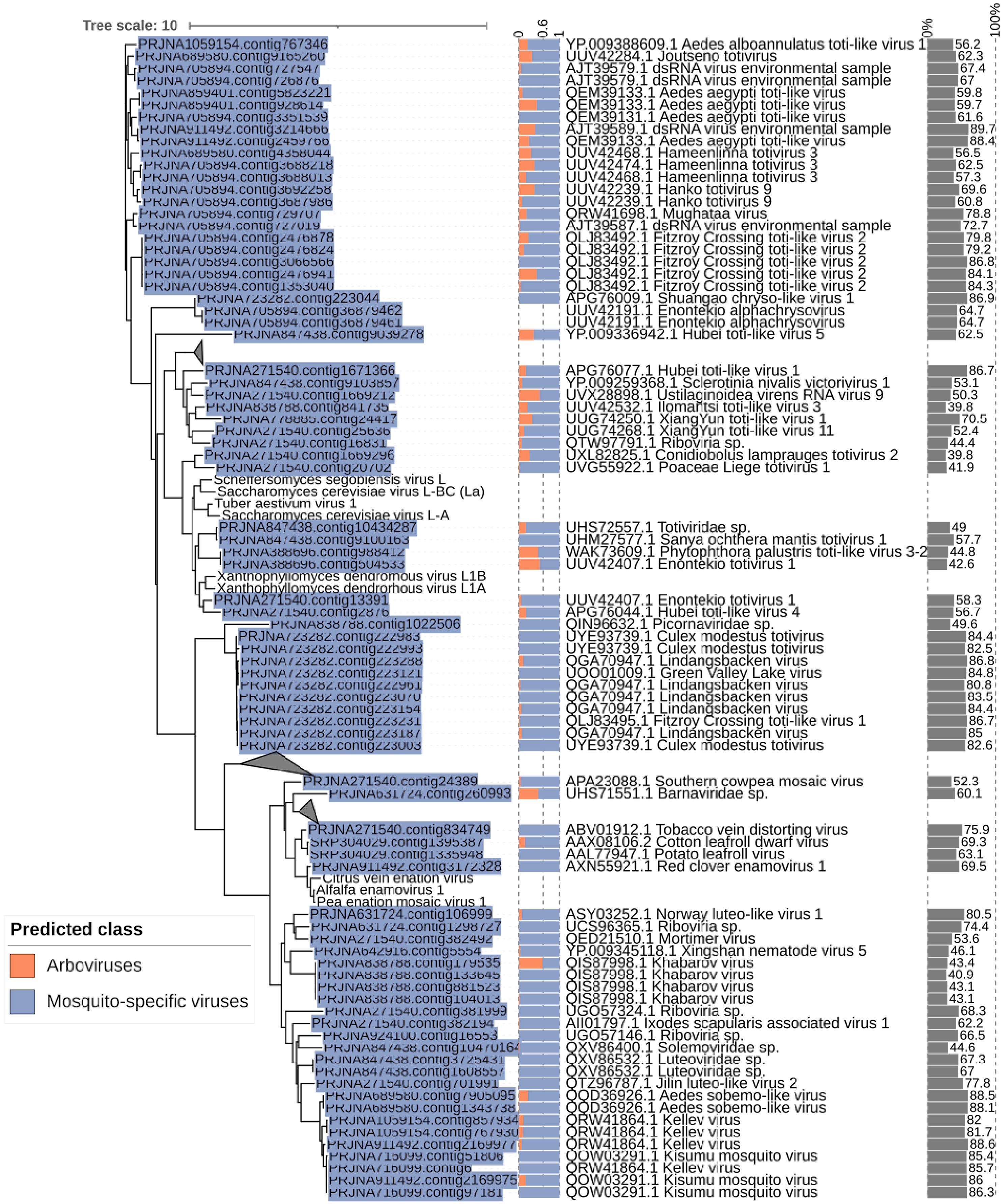

Figure 2. Phylogenetic tree for the RdRp_4 domain. The genera Trichomonasvirus (Totiviridae), Enamovirus (Solemoviridae), and Sobemovirus (Solemoviridae) were collapsed. Orange highlights putative novel arboviruses, while purple highlights mosquito-specific viruses.

3. RdRp domain: Mitovir_RNA_pol (PF05919)

For the Mitovir domain, we retrieved 40 representative sequences from complete RdRp proteins of viruses within the Birnaviridae family.

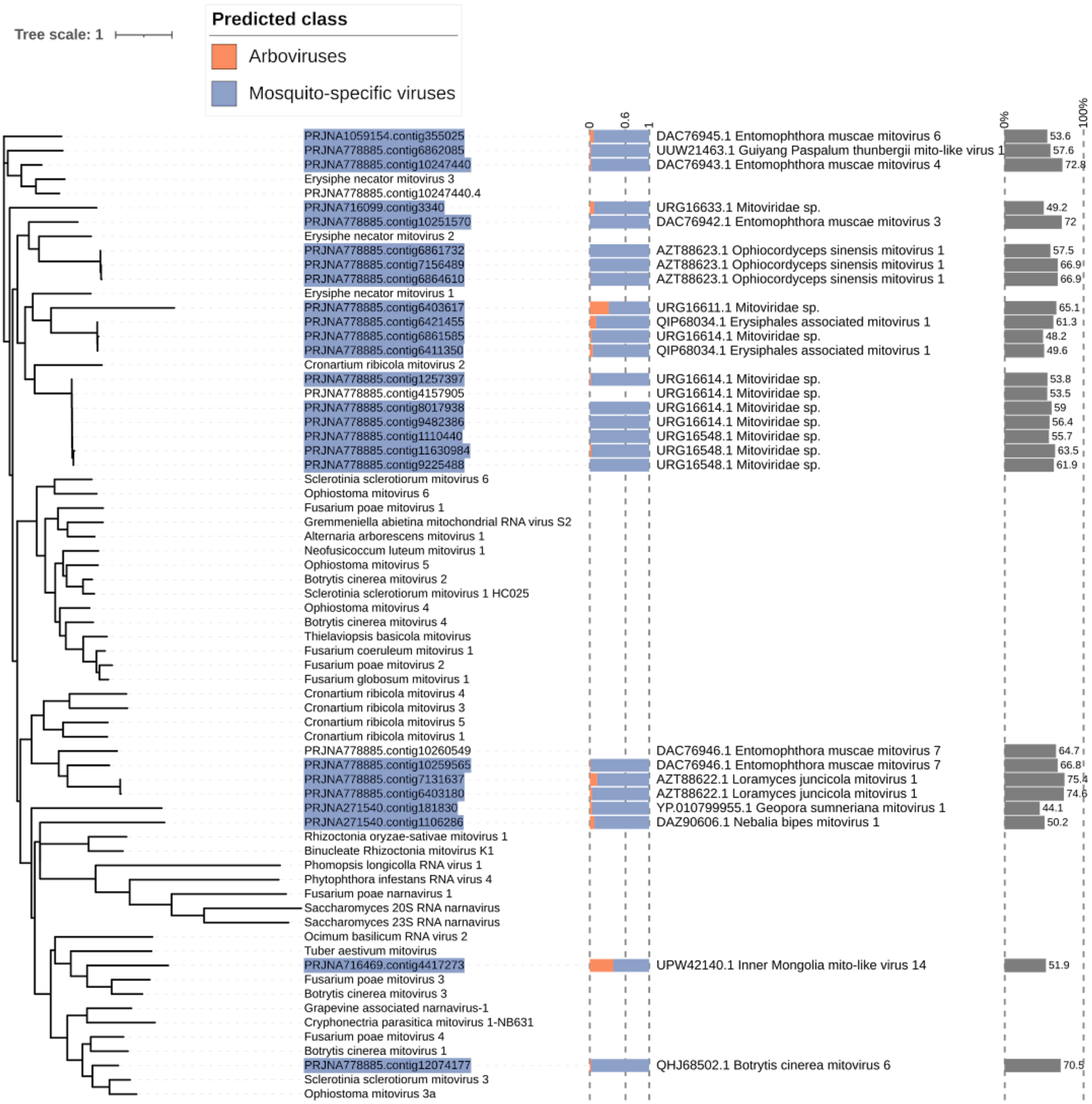

Figure 3. Phylogenetic tree for the Mitovir_RNA_pol domain. Putative novel Arboviruses are highlighted in orange, while putative novel Mosquito-specific viruses are represented in purple. The bar plot shows the probability score for the positive class per contig, emphasizing the score threshold set at 0.7. Additionally, the tree plot displays taxonomic information and identity scores based on the best hits against the NCBI non-redundant database.

4. RdRp domain: Flu_PB1 (PF00602)

This domain is related to the Orthomyxaviridae viral family, encompassing Influenza viruses. For this family, we retrieved 7 representative sequences.

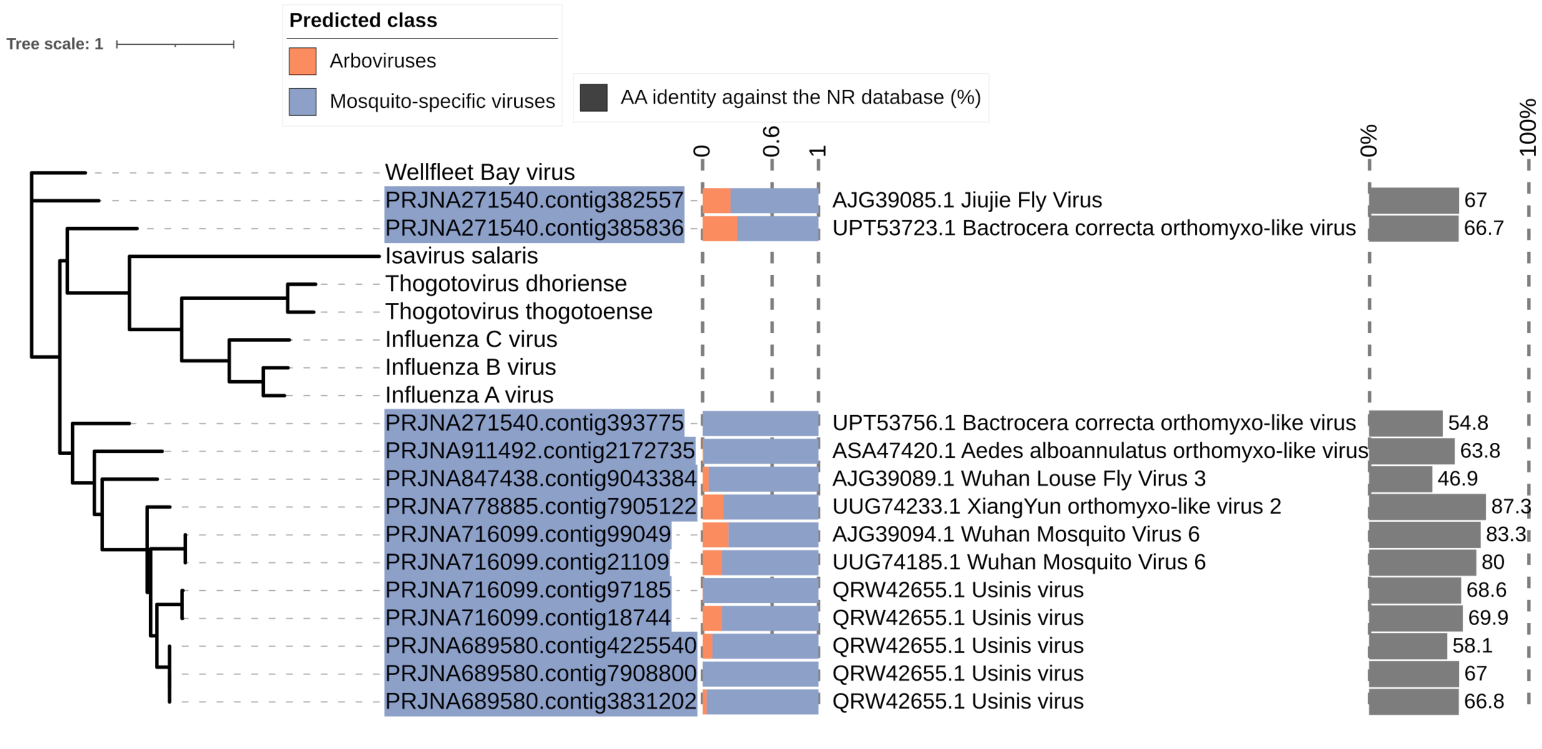

Figure 4. Phylogenetic tree for the FluPBI domain. Putative novel Arboviruses are highlighted in orange, while putative novel Mosquito-specific viruses are represented in purple. The bar plot shows the probability score for the positive class per contig, emphasizing the score threshold set at 0.6. The tree plot displays taxonomic information and identity scores based on the best hits against the NCBI non-redundant database.

5. RdRp domain: Mononeg_RNA_pol (PF00946)

This domain encompasses 10 families from the Mononegavirales order, as well as the family Chuviridae (from the Jingchuvirales order). We recovered 294 representative sequences for the families: Artoviridae, Bornaviridae, Filoviridae, Lispiviridae, Mymoviridae, Nyaviridae, Paramyxoviridae, Pneumoviridae, Rhabdoviridae, Sunviridae, Xinmoviridae, and Chuviridae. The representative sequences were aligned with 104 novel RdRp contigs. This phylogenetic tree provided comprehensive insights regarding the evolutionary relationships of 101 novel mosquito-specific viruses and 3 novel arboviruses with viral taxons from the Mononegavirales order (Figura 5).

The families Artoviridae, Bornaviridae, Filoviridae, Lispiviridae, Mymoviridae, Nyaviridae, Paramyxoviridae, Pneumoviridae, and Sunviridae, as well as the Rhabdoviridae genera Lyssavirus, Vesiculovirus, Ephemerovirus, Curiovirus, Siniperhavirus, Ledantevirus, Spirivirus, Cythorhabdovirus, and Orthorubulavirus, were collapsed due to the absence of novel RdRp contigs clustering with them. The families with the highest count of novel RdRp contigs were Rhabdoviridae (n. 56), Xinmoviridae (n. 18), and Chuviridae (n. 16).

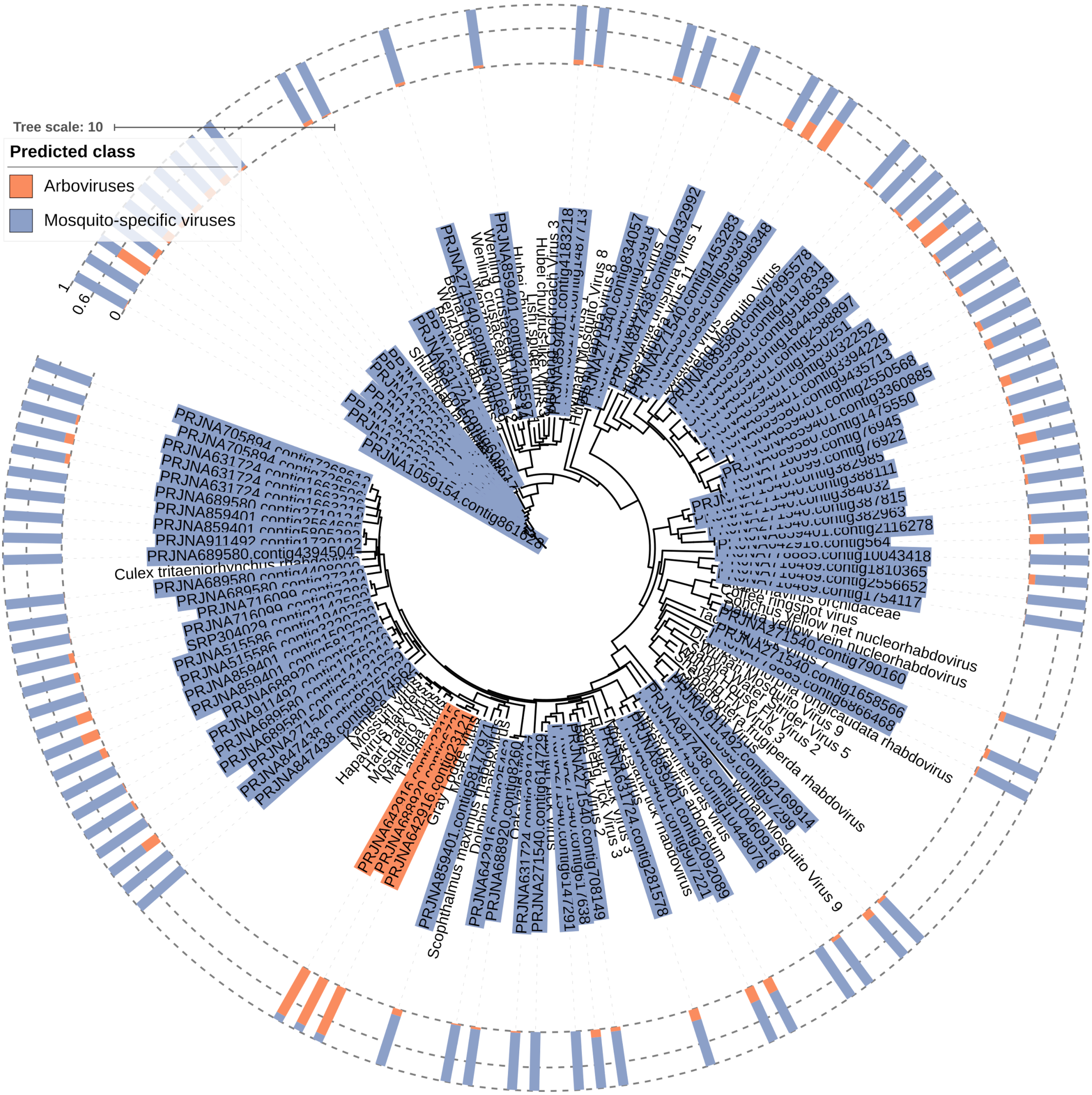

Figure 5. Phylogenetic tree for the Mononeg_RNA_pol (PF00946). Putative novel Arboviruses are highlighted in orange, while putative novel Mosquito-specific viruses are represented in purple. The bar plot shows the probability score for the positive class per contig, emphasizing the score threshold set at 0.7.

6. RdRp domain: RdRP_3 (PF00998)

This domain encompasses 52 representative sequences from the Tombusviridae and Nodaviridae viral families. Multiple genera did not form clusters with novel RdRp contigs, such as the Alphanecrovirus, Betacarmovirus, Gammacarmovirus, Panicovirus, Pelarspovirus, and were collapsed in the phylogenetic tree (Figure 6).

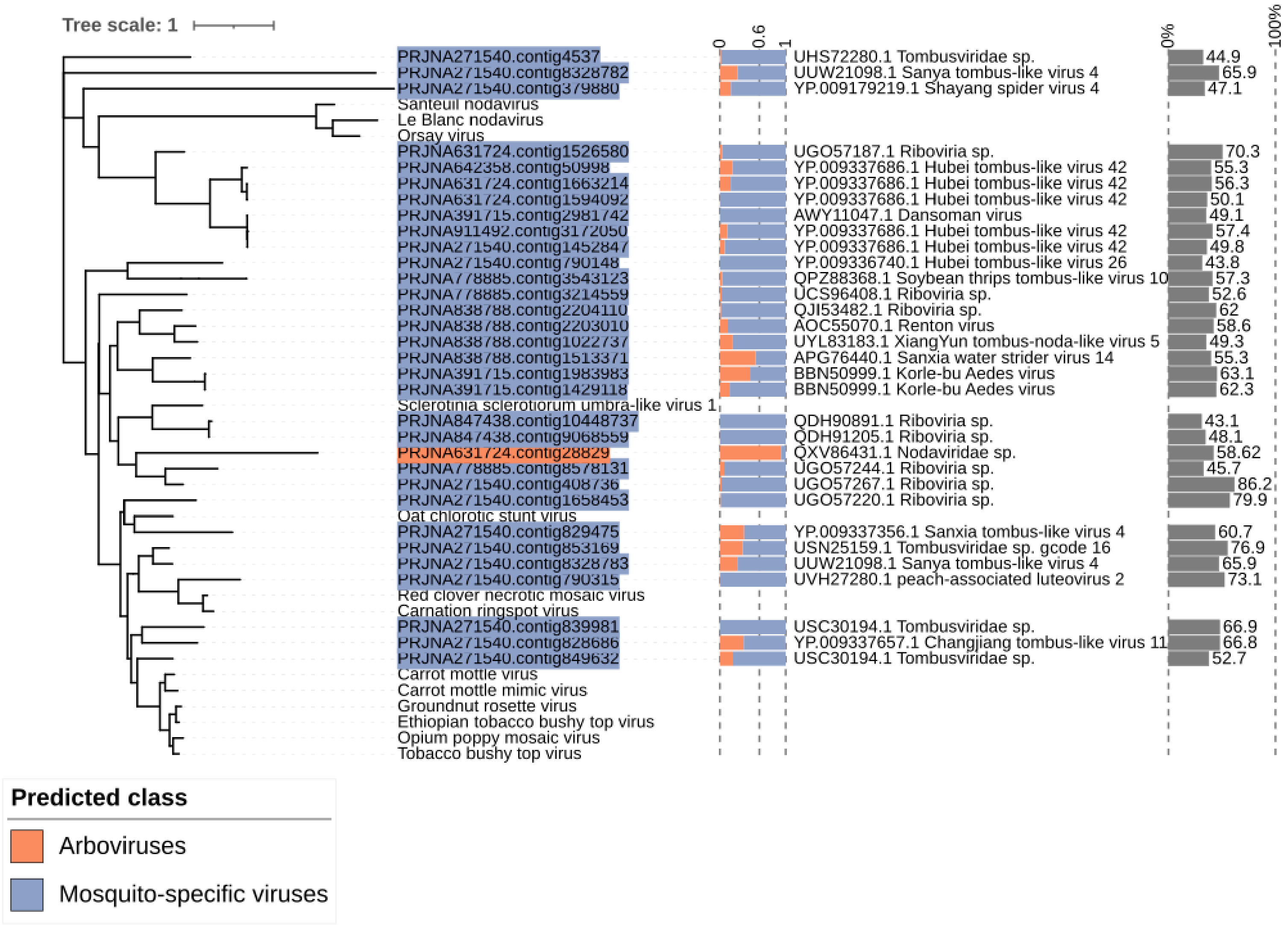

Figure 6. Phylogenetic tree for the RdRp_3 domain. Putative novel Arboviruses are highlighted in orange, while putative novel Mosquito-specific viruses are represented in purple. The bar plot shows the probability score for the positive class per contig, emphasizing the score threshold set at 0.7. The taxonomic information and identity scores are also displayed.

7. RdRp domain: RdRP_2 (PF00978)

This domain encompasses the Tymovirales order and the Hepe-Virga group. To build the phylogenetic tree we retrieved 290 representative RdRp sequences from the families: Alphaflexiviridae, Bromoviridae, Endornaviridae, Hepeviridae, Betaflexiviridae, Deltaflexiviridae, Gammaflexiviridae, Alphatetraviridae, Closteroviridae, Togaviridae, Virgaviridae, and the taxon Negevirus. In the tree (Figure 7), we collapsed the clades corresponding to the Alphaflexiviridae, Bromoviridae, Endornaviridae, Betaflexiviridae, Deltaflexiviridae, Gammaflexiviridae, Alphatetraviridae, Closteroviridae and Togaviridae.

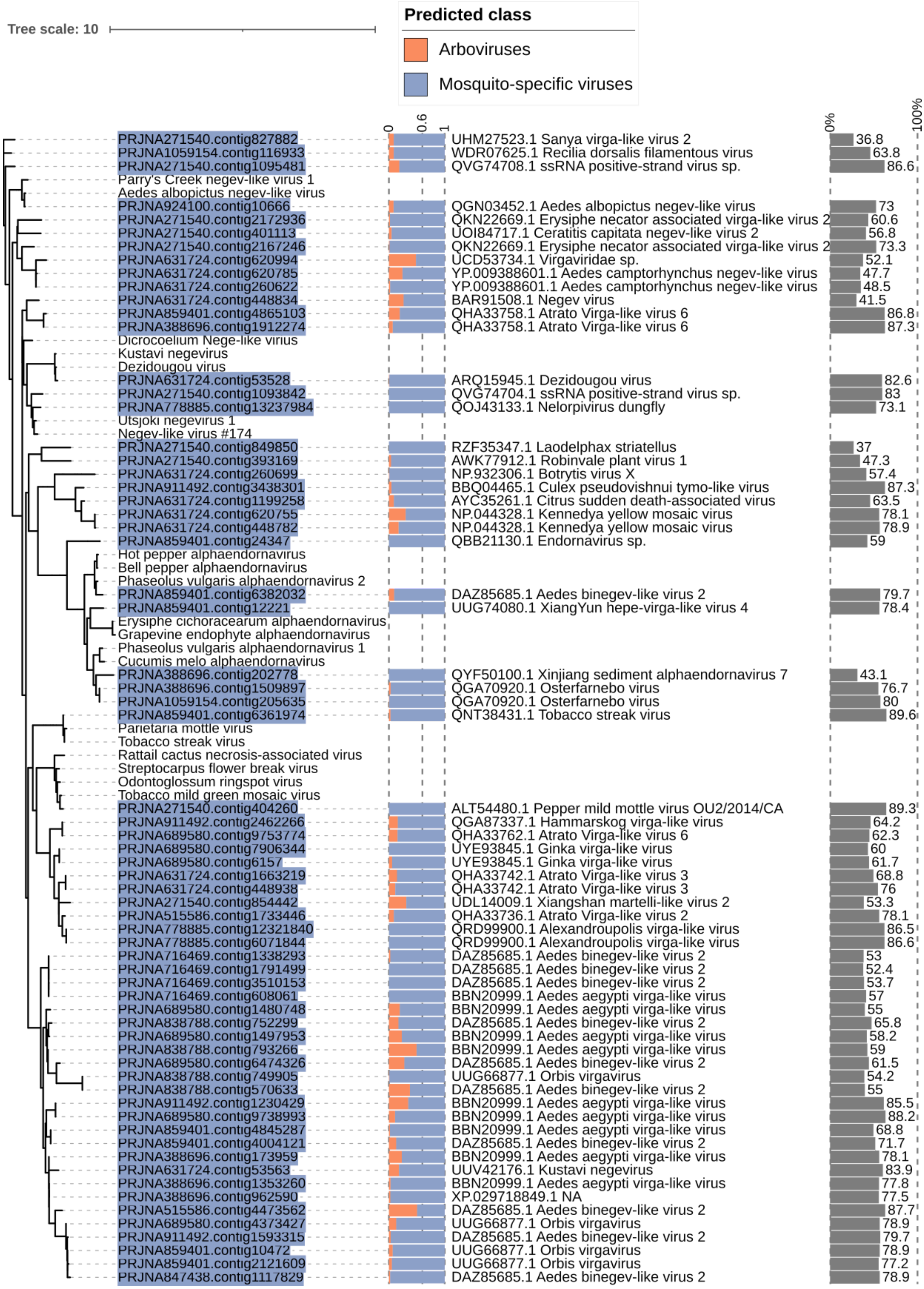

Figure 7. Phylogenetic tree for the RdRp_2 domain. Putative novel Arboviruses are highlighted in orange, while putative novel Mosquito-specific viruses are represented in purple. The bar plot shows the probability score for the positive class per contig, emphasizing the score threshold set at 0.7. Additionally, the tree plot displays taxonomic information and identity scores based on the best hits against the NCBI non-redundant database.

8. **RdRp domain: Flavi_NS5 (PF00972)**

We retrieved 140 representative sequences from the Flaviviridae family to build the phylogenetic tree (Figure 7). 62 of the novel RdRp contigs clustered with the Flavivirus genera.

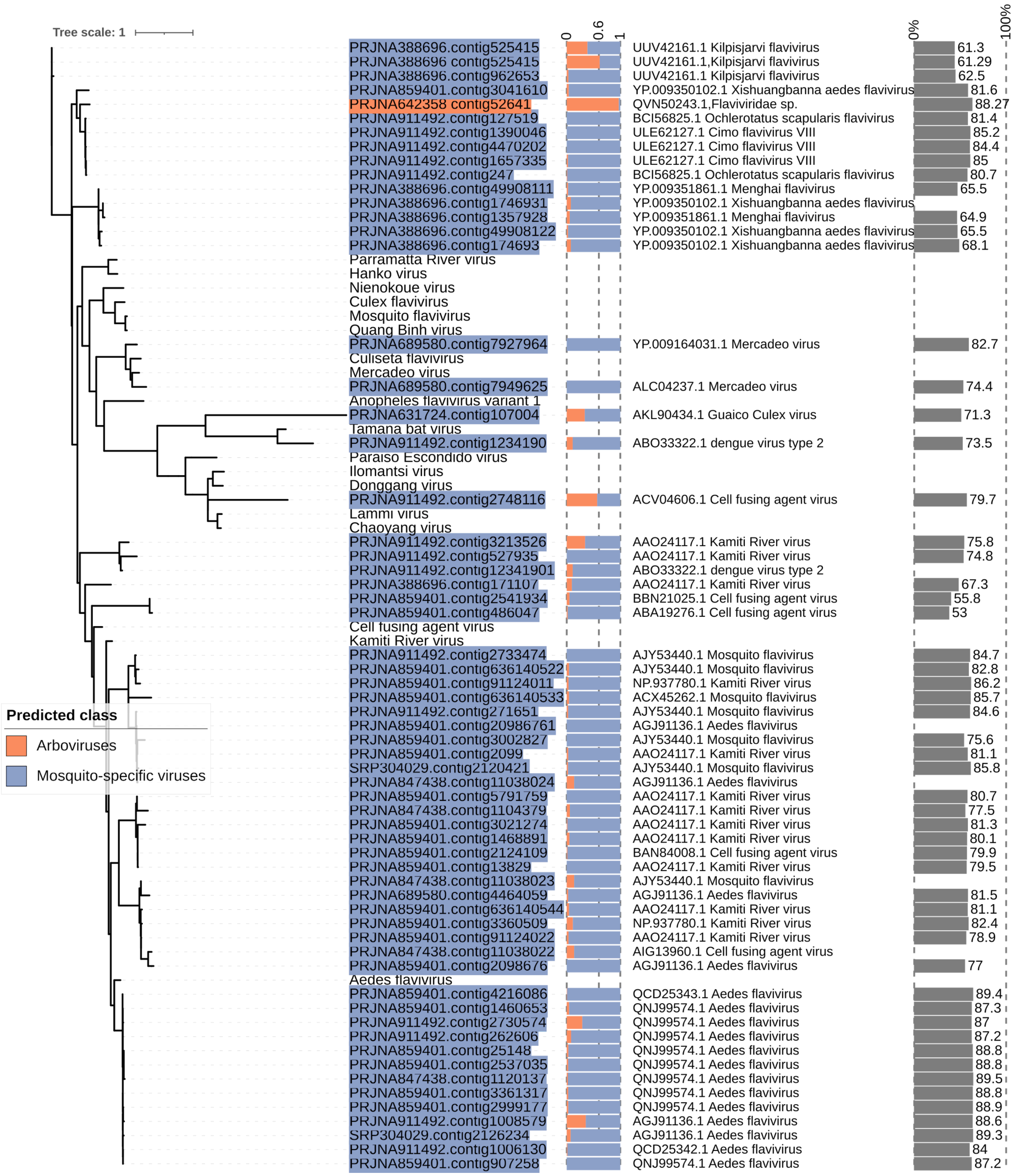

Figure 8. Phylogenetic tree for the Flaviviridae family. Putative novel Arboviruses are highlighted in orange, while putative novel Mosquito-specific viruses are represented in purple. The bar plot shows the probability score for the positive class per contig, emphasizing the score threshold set at 0.7 The tree plot displays taxonomic information and identity scores.

9. **RdRp domain: RdRP_1 (PF00680)**

This RdRp domain is related to the Picornavirales and Nidovirales viral orders. We retrieved 190 representative RdRp sequences from the families Arteriviridae, Dicistroviridae, Iflaviridae, Picornaviridae, Mesoniviridae, Coronaviridae, Secoviridae, Solinviviridae, Roniviridae, Caliciviridae, Marnaviridae, Polycipiviridae. In the phylogenetic tree (Figure 8), multiple genera and entire families collapsed due to no cluster with the novel RdRp contigs. The collapsed families were: Arteriviridae, Mesoniviridae, Coronaviridae, Secoviridae, Solinviviridae, Roniviridae, Caliciviridae, Marnaviridae, and, Polycipiviridae.

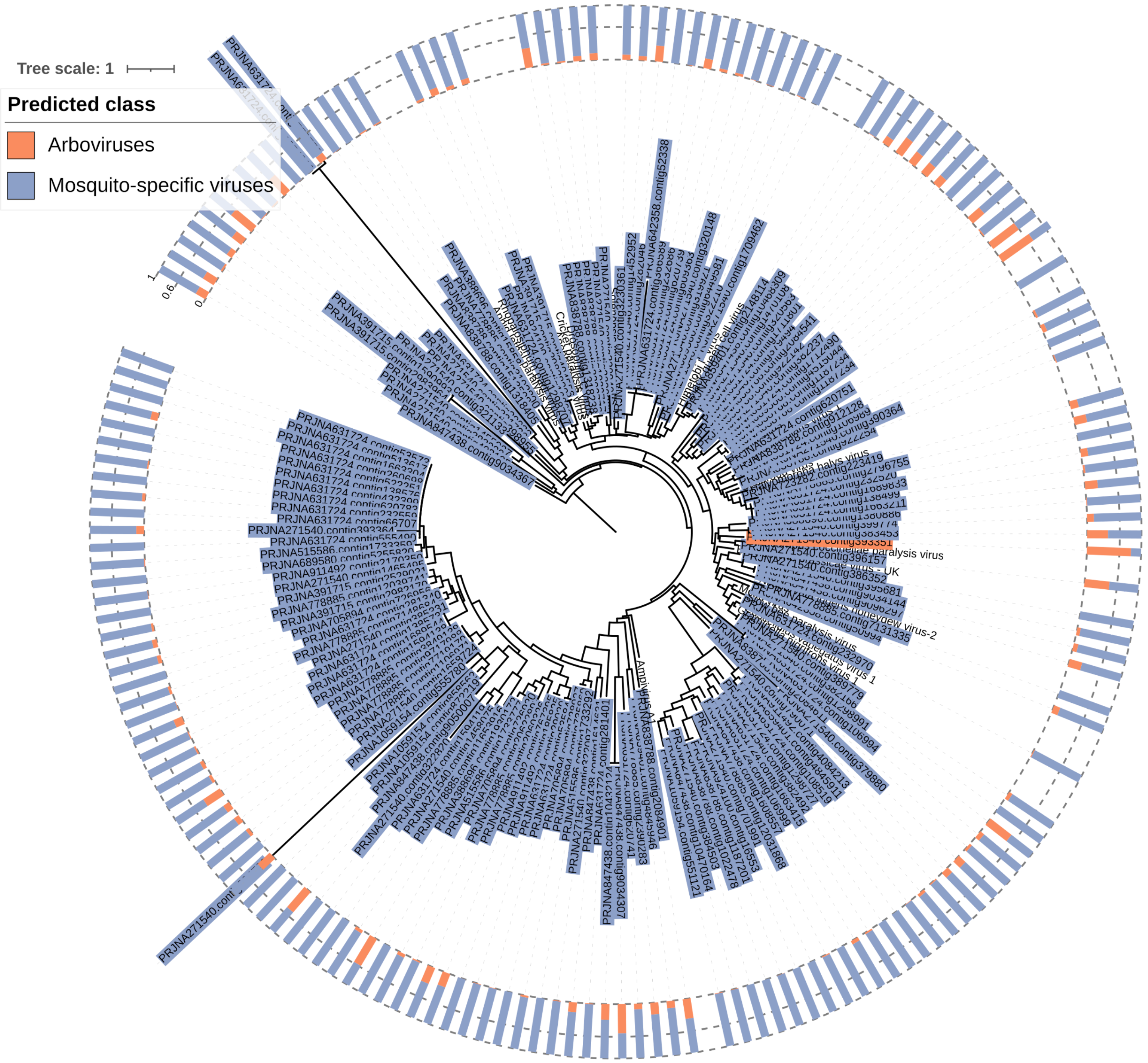

Figure 9. Phylogenetic tree for the RdRp1 domain. As the previous trees, all putative novel Arboviruses are highlighted in orange and putative novel Mosquito-specific viruses are represented in purple. The tree also displays the probability score for the positive class per contig, taxonomic information and identity scores based on the best hits against the NCBI non-redundant database.

10. RdRp domain: Bunya_RdRp (PF04196)

We retrieved 131 representative RdRp sequences from the Hantaviridae, Nairoviridae, Phasmaviridae, Wupedeviridae, Cruliviridae, Fimoviridae, Mypoviridae, Peribunyaviridae, Phenuiviridae, and Tospoviridae viral families. In our phylogenetic tree, only the Phasmaviridae, Peribunyaviridae, and Phenuiviridae clustered with novel RdRp contigs.

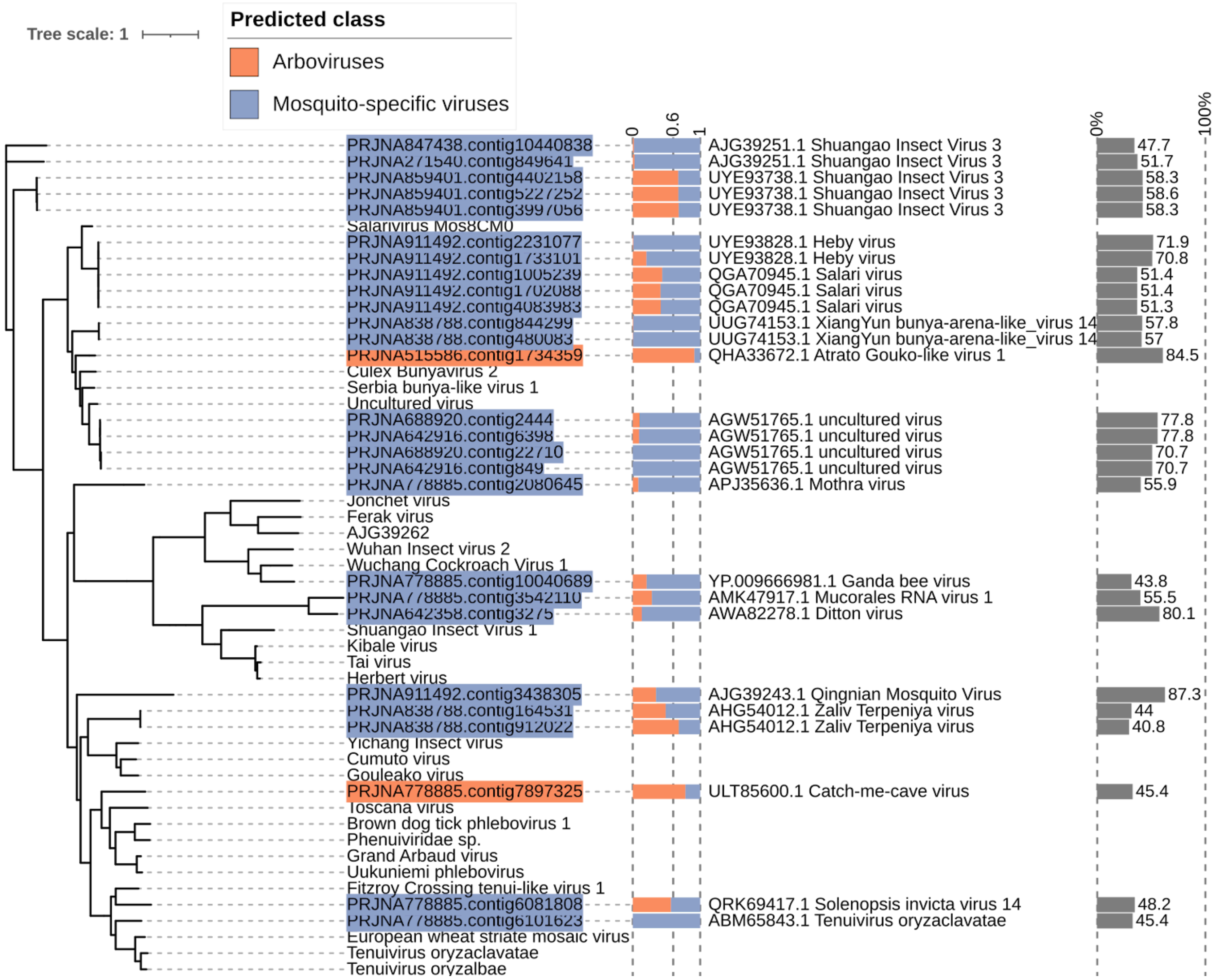

Figure 10. Phylogenetic tree for the BunyaRdRp domain. All putative novel Arboviruses are highlighted in orange, while putative novel Mosquito-specific viruses are represented in purple. The bar plot shows the probability score for the positive class per contig, emphasizing the score threshold set at 0.7. Additionally, the tree plot displays taxonomic information and identity scores based on the best hits against the NCBI non-redundant database.
