# Supplementary material for: Predicting novel mosquito-associated viruses from metatranscriptomic dark matter
Source: NAR Genom Bioinform. 2024 Jul 2;6(3):lqae077. doi: 10.1093/nargab/lqae077 (PMC11217672; doi:10.1093/nargab/lqae077)
Supplement: lqae077_Supplemental_Files [file lqae077_supplemental_files.zip › SM_TableS3_Andrade_et_al.pdf]

Supplementary Table 3. Overview of Viral Diversity and Sequence Retrieval Across Three Classes.

	Classes		
	Other viruses	Mosquito-Specific viruses (MSV)	Arboviruses
N. of viral species	7,808	298	92
N. of viral families	161	29	10
N. of retrieved sequences	1,040,303	22,906	509,531
N. remaining sequences	694,342 (66.7%)	8,227 (35%)	13,599 (2.6%)
Median lengths (bp)	10,836	8,824	10,179
