# Supplementary material for: Predicting novel mosquito-associated viruses from metatranscriptomic dark matter
Source: NAR Genom Bioinform. 2024 Jul 2;6(3):lqae077. doi: 10.1093/nargab/lqae077 (PMC11217672; doi:10.1093/nargab/lqae077)
Supplement: lqae077_Supplemental_Files [file lqae077_supplemental_files.zip › SM_TableS4_Andrade_et_al.pdf]

Supplementary Table 4. Feature extraction method selected for each fragment length from both steps. The predictive models exhibited varying percentages of True Negatives (TN), True Positives (TP), False Negatives (FN), and False Positives (FP), along with Area Under the ROC curve (AUC), Specificity, Sensitivity, and Precision.

Step 1: Mosquito-associated viruses (Positive) vs Other viruses (Negative)

Fragment length	TN (%)	TP (%)	FN (%)	FP (%)	mean AUC	mean Specificity	mean Sensitivity	mean Precision
10000 bp	55	44	0	1	0.99	0.99	0.98	0.99
5000 bp	50	49	0	1	0.99	0.98	0.98	0.99
3000 bp	48	49	1	2	0.92	0.89	0.90	0.90
1000 bp	45	43	5	7	0.89	0.90	0.85	0.81
500 bp	42	40	8	10	0.82	0.80	0.79	0.77

Step 2: Arboviruses (Positive) and Mosquito-specific viruses (Negative)

Fragment length	TN (%)	TP (%)	FN (%)	FP (%)	mean AUC	mean Specificity	mean Sensitivity	mean Precision
10000 bp	51	48	0	1	0.99	0.99	0.99	0.99
5000 bp	50	45	1	4	0.95	0.90	0.91	0.92
3000 bp	49	45	1	5	0.91	0.85	0.90	0.88
1000 bp	43	39	7	11	0.79	0.80	0.79	0.79
500 bp	42	35	10	13	0.75	0.75	0.72	0.70
