# Supplementary material for: Predicting novel mosquito-associated viruses from metatranscriptomic dark matter
Source: NAR Genom Bioinform. 2024 Jul 2;6(3):lqae077. doi: 10.1093/nargab/lqae077 (PMC11217672; doi:10.1093/nargab/lqae077)
Supplement: lqae077_Supplemental_Files [file lqae077_supplemental_files.zip › SM_TableS5_Andrade_et_al.pdf]

Supplementary Table 5. Functional annotation for the 641 novel RdRp contigs identified in this study, using the probability score threshold 0.7. The contigs vary in functional annotation of RdRp domains, the number of novel RdRp contigs per domain, and the distribution of contigs among different classes.

Taxonomic assessment	RdRp domain	N. of novel RdRp contigs	N. of Other viruses	N. of Mosquito-specific viruses	N. of Arboviruses
Picornavirales-like and Nidovirales-like	RdRP_1 (PF00680)	184	4	171	1
Tymovirales-like and Hepe-Virga-like	RdRP_2 (PF00978)	69	11	58	0
Tombusviridae-like and Nodaviridae-like	RdRP_3 (PF00998)	59	2	56	1
Toti-, Luteo-, and Sobemoviridae-like	RdRP_4 (PF02123)	91	2	89	0
Reoviridae-like	RdRP_5 (PF07925)	0	-	-	-
Birnaviridae-like	Birna_RdRp (PF04197)	2	1	1	0
Flaviviridae-like	Flavi_NS5 (PF00972)	66	3	62	1
Narnaviridae-like	Mitovir_RNA_pol (PF05919)	28	2	26	0
Bunyavirales-like	Bunya_RdRp (PF04196)	34	6	26	2
Arenaviridae-like	Arena_RNA_pol (PF06317)	0	-	-	-
Mononega- and Chuviridae-like	Mononeg_RNA_pol (PF00946)	97	4	90	3
Orthomyxoviridae-like	Flu_PB1 (PF00602)	14	1	13	0
